# Animacy processing by distributed and interconnected networks in the temporal cortex of monkeys

**DOI:** 10.3389/fnbeh.2024.1478439

**Published:** 2024-12-13

**Authors:** Rizal Ichwansyah, Keigo Onda, Jun Egawa, Takeshi Matsuo, Takafumi Suzuki, Toshiyuki Someya, Isao Hasegawa, Keisuke Kawasaki

**Affiliations:** ^1^Department of Neurophysiology, Niigata University School of Medicine, Niigata, Japan; ^2^Department of Psychiatry, Niigata University School of Medicine, Niigata, Japan; ^3^Department of Neurosurgery, Tokyo Metropolitan Neurological Hospital, Tokyo, Japan; ^4^Center for Information and Neural Networks, National Institute of Information and Communications Technology, Osaka, Japan

**Keywords:** categorization, animacy, inferior temporal cortex, prefrontal cortex, electrocorticography, superior temporal sulcus, granger causality

## Abstract

Animacy perception, the ability to discern living from non-living entities, is crucial for survival and social interaction, as it includes recognizing abstract concepts such as movement, purpose, and intentions. This process involves interpreting cues that may suggest the intentions or actions of others. It engages the temporal cortex (TC), particularly the superior temporal sulcus (STS) and the adjacent region of the inferior temporal cortex (ITC), as well as the dorsomedial prefrontal cortex (dmPFC). However, it remains unclear how animacy is dynamically encoded over time in these brain areas and whether its processing is distributed or localized. In this study, we addressed these questions by employing a symbolic categorization task involving animate and inanimate objects using natural movie stimuli. Simultaneously, electrocorticography were conducted in both the TC and dmPFC. Time-frequency analysis revealed region-specific frequency representations throughout the observation of the movies. Spatial searchlight decoding analysis demonstrated that animacy processing is represented in a distributed manner. Regions encoding animacy information were found to be dispersed across the fundus and lip of the STS, as well as in the ITC. Next, we examined whether these dispersed regions form functional networks. Independent component analysis revealed that the spatial distribution of the component with the most significant animacy information corresponded with the dispersed regions identified by the spatial decoding analysis. Furthermore, Granger causality analysis indicated that these regions exhibit frequency-specific directional functional connectivity, with a general trend of causal influence from the ITC to STS across multiple frequency bands. Notably, a prominent feedback flow in the alpha band from the ITC to both the ventral bank and fundus of the STS was identified. These findings suggest a distributed and functionally interconnected neural substrate for animacy processing across the STS and ITC.

## 1 Introduction

Animacy perception refers to the ability to distinguish between living and non-living entities based on their behavior and appearance. This cognitive ability is crucial because it enables organisms to prioritize and respond appropriately to biologically relevant stimuli, such as potential predators or conspecifics, which is essential for survival and social interaction. This ability is suggested to exist not only in humans but also in non-human primates, including monkeys (Fabre-Thorpe et al., 1998; Hauser, 1998; Tsutsumi et al., 2012; Yetter et al., 2021).

Functional magnetic resonance imaging (fMRI) studies in both humans and monkeys have identified the ventral temporal cortex (VTC) as a key region for processing animacy information, contributing to various aspects of perception, including visual categorizability (Bell et al., 2009; Chao et al., 1999; Kriegeskorte et al., 2008; Naselaris et al., 2012; Pinsk et al., 2005) and agency (Thorat et al., 2019). Single units and intrisic optical imaging (IOI) studies in monkeys suggest that columnar/patch and domain like functional structures for encoding shape information, including animacy attribution such as face and body parts in the gyri, the inferior temporal cortex (ITC) (Desimone et al., 1984; Kiani et al., 2007; Kriegeskorte et al., 2008; Sato et al., 2013; Tanaka, 1996; Tsao, 2014), while biological motion processing appears to be handled by regions in the superior temporal sulcus (STS) (Jastorff et al., 2012; Nelissen et al., 2006; Oram and Perrett, 1994, 1996; Vangeneugden et al., 2011).

Additionally, the prefrontal cortex is also critical for animacy perception. In particular, the dorsomedial prefrontal cortex (dmPFC) is involved in cognitive processing for understanding one’s own and others’ intentions and agency, and it plays important roles in animacy perception within social contexts through human fMRI studies (Amodio and Frith, 2006; Wheatley et al., 2007).

However, due to the limited temporal resolution of MRI and IOI, as well as the sparse sampling and restricted spatial coverage of single-cell recording methods, it remains unclear how animacy perception is dynamically encoded over time in these two brain regions, the temporal cortex (TC), and the dmPFC, and how this encoding is distributed or localized within these areas. More specifically, whether the encoding is spread across the TC and dmPFC, within specific areas like the ITC and STS within the TC, or confined to particular subregions. Furthermore, the physiological substrates underlying these spatial encodings have yet to be elucidated.

To address these questions, we employed a symbolic categorization task while conducting high-density ECoG recordings across the TC including both the ITC and STS as well as the dmPFC. The task involved natural movie stimuli depicting animate and inanimate agents, rich in shape and motion elements. This approach, unlike previous studies that used static images (Chao et al., 1999; Kiani et al., 2007; Kriegeskorte et al., 2008) or point-light displays (Oram and Perrett, 1994), allowed us to examine the integration of shape and motion and the processing of agency.

Using linear decoding, we analyzed the time-frequency dynamics of animacy information encoding within each brain region. Spatial searchlight decoding analysis allowed us to assess how distributed the animacy information was encoded, identifying distributed representations in both the ITC and STS. To determine whether these distributed representations formed functional subnetworks, we applied independent component analysis (ICA). Granger causality analysis further revealed frequency-specific directional patterns of functional connectivity within the subnetwork.

## 2 Materials and methods

### 2.1 Animal care and use

Two Japanese monkeys (*Macaca fuscata*), referred to as monkey P (male, 6.5 kg) and monkey J (female, 4.7 kg), were used in this study. The monkeys were provided by National BioResource Project (NBRP) “Japanese Monkeys,” Japan. They were housed in standardized primate cages and adequately given primate food supplemented with fruits and vegetables. All experimental procedures involving animals were carried out in accordance with the Guide for the Care and Use of Nonhuman primates in Neuroscience Research (The Japan Neuroscience Society; https://www.jnss.org/en/animal_primates). The experimental protocol was approved by the Institutional Animal Care and Use Committee of Niigata University (Permission number SA00218).

### 2.2 General surgical procedures

The ECoG grids were implanted under aseptic conditions as described previously (Matsuo et al., 2011). After premedication with ketamine (50 mg/kg) and medetomidine (0.03 mg/kg), each animal was intubated with an endotracheal tube subsequently was anesthetized with isoflurane (1.5–3.0%) under mechanical ventilation (A.D.S. 1000, Engler Engineering Corp., FL, United States) throughout the surgery. The venous line was secured using lactated Ringer’s solution, and ceftriaxone sodium hydrate (100 mg/kg) was administered as a prophylactic antibiotic. Their body temperature was maintained at 37°C during the surgery by using an electric heating mat. A vacuum fixing bed (Vacuform, B.u.W.Schmidt GmbH, Garbsen, Germany) was used to maintain the position of the body. Vital signs (hearth rate and rhythm, respiratory rate, non-invasive blood pressure, haemoglobin saturation) were continuously monitored (Surgi Vet, Smiths Medical PM Inc., London, UK) during anesthesia. Postoperatively, the monkeys were given postsurgical analgesic (ketoprofen, 1 mg/kg/day) for at least 3 days and the antibiotic was continued for 1 week after the surgery.

### 2.3 Electrode array

We custom-designed and fabricated a sheet-type, minimally invasive electrode grids to measure ECoG signals from the TC and dmPFC of monkeys. The arrays were fabricated on a 20 μm-thick flexible Parylene-C film using micro-electro-mechanical systems technology. The basic structures and fabrication processes of the probe have been previously described elsewhere (Matsuo et al., 2011; Toda et al., 2011; Kaiju et al., 2017). The probe arrays included 8 × 16 electrodes, which covered 18.5 mm x 38.5 mm for recording surface potentials from the ITC, and 8 × 8 electrodes, which covered 18.5 mm x 18.5 mm for recording surface potentials from the dmPFC. Each square contact had a gold surface measuring 1 mm x 1 mm. The center-to-center distances between adjacent contacts were 2.5 mm. Electrode impedances typically ranged from 3–5 kΩ at 1 kHz.

### 2.4 Electrode implantation

**Figure 1:**
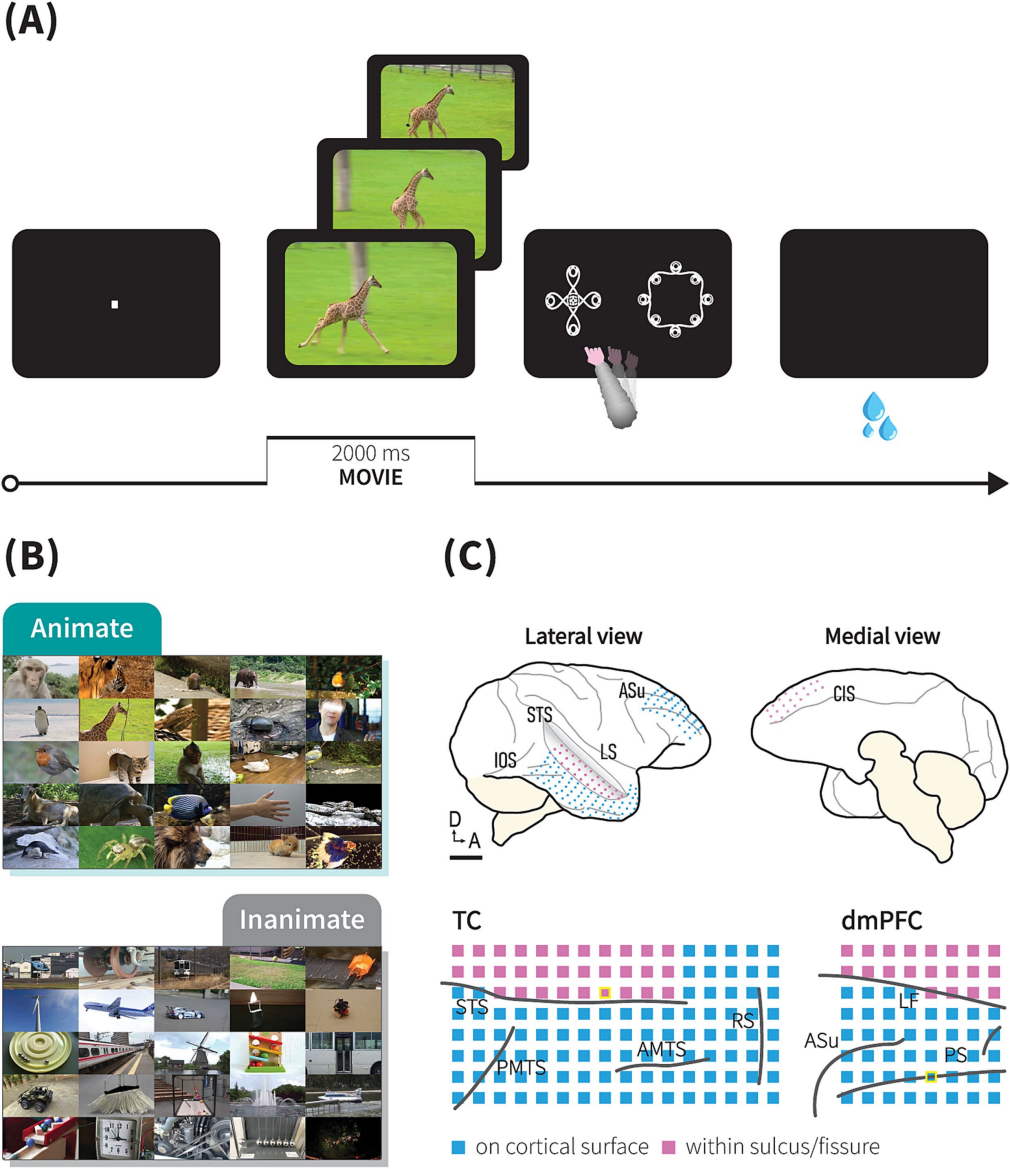
Behavioral task and ECoG recordings. **(A)** Task trial sequence. Following central fixation, a 2-s movie was presented, followed by the appearance of two symbols, during which monkeys needed to choose a symbol representing the movie category by touching the screen. In this trial sequence example, the animate and animate symbols appeared on the left and right sides, respectively. If monkeys chose the correct symbol, juice reward was immediately delivered. **(B)** Examples of movie stimuli depicting moving living (animate) or non-living objects (inanimate). See method for further details. **(C)** ECoG electrode array locations. Upper: schematic drawings of a macaque brain showing location of implanted electrodes in monkey P. Lower: detailed location of electrodes relative to sulci and fissure (STS, superior temporal sulcus; AMTS, anterior middle temporal sulcus; IOS, inferior occipital sulcus; PMTS, posterior middle temporal sulcus; RS, rhinal sulcus; PS, principal sulcus; ASu, upper limb arcuate sulcus; CIS, cingulate sulcus; LF, longitudinal fissure). Blue and purple squares represent electrodes implanted on cortical surfaces and within a sulcus/fissure, respectively. Scale bars, 10 mm. Location of channels presented in Figure 2 are marked with yellow border.

To determine the implanted areas, we used preoperative magnetic resonance imaging for stereotactic guidance. The detailed procedures for microscopic neurosurgery for ECoG grid implantation under aseptic conditions have been described elsewhere (Matsuo et al., 2011). Briefly, the skull was fixed with a three-point fastening device (Integra Co., NJ, United States) with a custom-downsized attachment for macaques. The implantation started with removing skin, galea aponeurotica, and muscle over the implanted areas. Craniotomy and durotomy were performed using microscope (Ophthalmo-Stativ S22, Carl Zeiss Inc., Oberkochen, Germany) with a CMOS color camera (TS-CA-130MIII, MeCan Imaging Inc., Saitama, Japan). We placed the grids on the cortical surface of the TC and dmPFC with a number of electrodes placed into sulci and fissure as shown in Figure 1C and Supplementary Figure S2. A reference electrode was placed close to the grids in the subdural space facing the dura. After the grids were implanted, the dura was closed with watertight suturing to prevent cerebrospinal fluid leakage. The electrode lead, micro connectors (Omnetics, MN, United States), and a custom-made connector chamber made of polyetherimide (Vivo, Hokkaido, Japan) were fixed onto the bone with dental acrylic. Electrode locations in the animals’ brains were confirmed postmortem.

**Figure 2:**
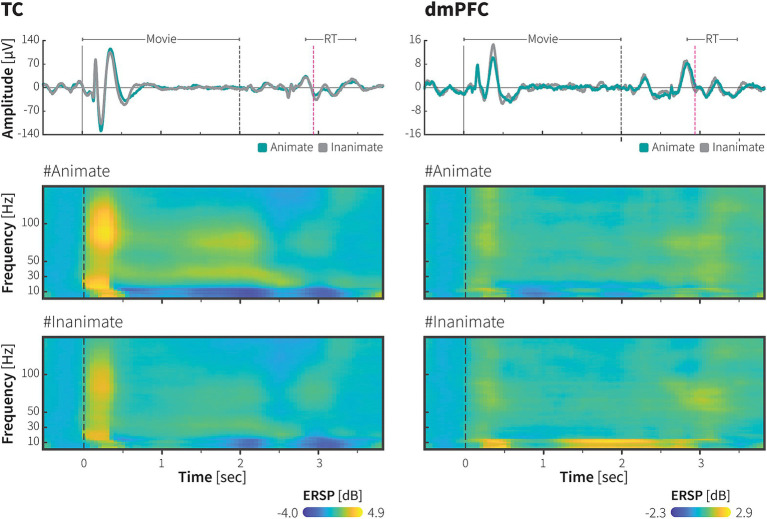
ECoG responses during animacy category task in the TC and dmPFC. Event-related potential (ERP; upper panel) and event-related spectral perturbation (ERSP; middle and lower panels) from single electrodes in the TC and dmPFC of monkey P, aligned to the onset of the movie stimuli during the symbolic animate/inanimate task, with each response averaged over 1,667 trials. In the upper panels, green and grey-color lines indicate LFPs during animate and inanimate trials, respectively. Black dashed lines indicate the end of the movie presentation, immediately followed by the onset of symbols. Magenta dash lines and horizontal lines above the lines indicate median RT and the range of RT across 1,667 trials (median 910 ms, range: 806 ms – 1,450 ms), respectively. Color scale bars indicate log-transformed spectral power ratio normalized by the pre-stimulation power (See Methods for details). RT, reaction time.

### 2.5 Behavioral tasks

We trained the monkeys to perform an animate/inanimate categorization task (Figures 1A,B). In contrast to the go/no-go task used in the previous studies (Fabre-Thorpe et al., 1998; Yetter et al., 2021), our task dissociated behavioral responses from the category depicted in the movie, therefore requiring the monkeys to engage cognitive processes, such category abstraction, during category choice selection. The stimuli were presented on a 15-inch cathode ray tube (CRT) monitor (NEC, Tokyo, Japan) and a 13.5-inch- infrared touch panel were attached on the front side of the monitor (Caroll Touch, CA, United States). The distance between touch screen panel to monkeys’ eyes was around 14.5 cm. To capture eye movement, we monitored eye position using an infrared pupil-position monitoring system (iRecHS2, Matsuda et al., 2017) at sampling rate of 60 Hz. Each trial began when the monkey held down a lever after an auditory ‘start’ tone was sounded. A fixation point (0.1° x 0.1° in visual angle, square) appeared at the center of monitor, and the animal was required to maintain its gaze within ±1.5° of the fixation point until a movie started (500 ms); whenever the monkeys aborted fixation, the trial terminated without a reward. After the fixation, a 2-s-long movie clip depicting moving animals (animate), or movements of non-living objects (inanimate) was played. Immediately after the movie ended, two symbols indicating each category were presented at a random location after a one-frame delay (16.6 ms) and monkeys were rewarded if they released the lever and selected the correct symbol by touching the monitor. In correct trials, following the reward juice delivery, a sound indicating a correct answer was played. In error trials, without the juice delivery, a sound indicating an incorrect answer was played. An aborted trial was defined as either a lever release before the movie ended, fixation breaks, or when the estimated gaze exceeded the monitor’s range during movie presentation. An inter-trial interval of 2 s was inserted before the next ‘start’ tone. Only the data from completed trials were included in the analysis.

The stimuli were presented with an x86 PC running a custom-written OpenGL-based stimulation program under Windows XP. Behavioral control for the experiments was maintained by a network of interconnected PCs running QNX-real-time operating system (QSSL, ON, Canada), which controlled the timing and synchronization. Data was monitored online and stored on a PC-based system (NSCS, Niigata, Japan) or on System 3 real-time signal processing systems (Tucker Davis Technologies, FL, United States).

### 2.6 Stimulus movie

The stimulus movies were used either from movies uploaded on a video platform, Youtube, or movies recorded by our laboratory members. The videos were then edited into 2-s clips using MoviePy.^1^ Finally, we selected clips in which only moving animals or non-living objects were clearly shown.

### 2.7 Learning procedures

The monkeys were initially trained using a dataset consisting of six movies (three movies for each category). We measured the correct response rate for each block of 30 trials for each movie. Once the monkeys achieved the learning criterion of maintaining an accuracy rate of over 80% for five consecutive blocks, a new set of six movies was added as test stimuli. This new set included both the six trained stimuli and six entirely new stimuli. To test whether the monkeys learned the symbolized animate/inanimate category, we computed monkey responses to the first encountered stimuli. Following the first encounter test, training continued, and once the accuracy criterion of 80% was achieved for these 12 stimuli, another set of six new test stimuli was introduced. The stimuli consisted of a total of 18 stimuli (12 trained movies +6 novel movies). Similarly, after each set of training, a new set of six stimuli was introduced for testing. In total, monkey P completed learning and testing over 10 sets, while monkey J completed 7 sets. Sets 1 to 7 were shared between them, with identical learning and testing procedures. Additionally, only monkey P underwent further testing and training using trial-unique stimulus sets. In each trial-unique set, there were 84–150 novel stimuli per category, with each movie stimulus presented only once during the training/test phase. We assumed that if the monkey was relying on associative features to perform the task with high accuracy, presenting the unique-trial dataset should cause a decrease in categorization performance to chance. The recording session began after the training/test phase was completed. In the recording sessions, we used the learned 768 movies, including the trial-unique datasets, for monkey P and 42 movies for monkey J. The recall and precision rates were calculated using animate stimuli as relevant stimuli.

After preprocessing procedure (see ECoG recordings), the analyzed dataset included 538 stimuli (271 animate movies and 267 inanimate movies) across 1,667 trials in monkey P’s dataset and 42 stimuli (21 movies in each category) across 2,109 in monkey J’s dataset.

### 2.8 ECoG recordings

ECoG recordings were conducted in an electrically shielded, sound-attenuated chamber. The signals were amplified and a band-pass filtered (Butterworth, 0.7–300 Hz) using PZ2 PreAmp (Tucker Davis Technologies, FL, United States), sampled digitally, and stored on hard-disk drives at a sampling rate of 2034.5 kHz.

Data processing was performed using custom MATLAB codes (MathWorks, MA) with EEGLAB toolbox (https://eeglab.org/, Delorme and Makeig, 2004). ECoG signals were segmented, from 0.7 s before to 4 s after the onset of movie stimuli. To prevent aliasing effect during resampling, a low-pass filter at 220 Hz was initially applied, followed by downsampling the signal to 500 Hz with the cutoff frequency at 400 Hz and the transition bandwidth at 200 Hz, and subsequently applying a high-pass filter at 1 Hz. We employed the ‘Zapline-plus’ plugin, available within EEGLAB, to remove 50-Hz line noise from the ECoG signals (de Cheveigné, 2020; Klug and Kloosterman, 2022). Trials containing artifacts in the signals were manually identified and rejected.

Signals from each channel were re-referenced by subtracting the average values of electrodes in the same array. Noise components were eliminated using independent component analysis. Detailed procedures for noise components identification are shown in the independent component analysis section. Time-frequency analyses were calculated and visualized by using the Chronux 2.12 toolbox (http://chronux.org/, Bokil et al., 2010), implemented in MATLAB. Event-related spectral perturbation (ERSP) was calculated using a multitaper power spectral density estimation. For the high-resolution ERSP, the power spectra in the frequency range of 1.5–150 Hz were computed individually for each channel and trial, using 6 Slepian tapers and a time-bandwidth product of 4. We used a moving window size of 400 ms and a step size of 50 ms, giving a frequency resolution of 2 Hz (totally, there were 87 time points and 76 frequency points). For the low-resolution ERSP, time-frequency analyses were calculated by using FieldTrip toolbox (http://fieldtriptoolbox.org/, Oostenveld et al., 2011). The power spectra for six frequencies (3, 8, 12, 16, 58, and 89 Hz) were computed using a multitaper with a 500 ms moving window and a 250 ms step size (resulting in 6 frequency points and 17 time points). The ERSP was normalized using single-trial baseline normalization. Baseline period was from of −700 ms to 0 before stimulus onset. The normalization involved dividing each time-frequency point by the average spectral power in the pre-stimulus baseline period at the same frequency, then we computed the trial average spectral power (Grandchamp and Delorme, 2011). We transformed absolute ERSP measure into a decibel (dB) scale.

Once all the recording experiments were completed, the animals were administered an overdose of sodium pentobarbital and transcardially perfused with 4% paraformaldehyde in 0.1 M phosphate buffer (pH 7.4). Electrode locations in the animals’ brains were determined postmortem.

### 2.9 Decoding analysis

In the neuronal population decoding (for both resolutions), channels were grouped into two populations, i.e., ITC and dmPFC, based on the cortical area. Non-normalized power spectral (or spectrum) at the same time-frequency point were pooled and concatenated across trials to build the feature vectors for each area. The feature matrix of each time-frequency point was number of trials x 128 for the ITC and number of trials x 64 for the dmPFC. We then calculated decoding accuracy for each time-frequency point. We assessed the decoding accuracy using a 5-fold cross-validation procedure to avoid overfitting in the classifier model. Specifically, the trials were split into five subsets, and we used four subsets (training dataset) to train a linear support vector machine (SVM) classifier to discriminate category based on the power spectral. In doing so, we used the default hyperparameters as defined in the function. Finally, we quantified the classifier’s accuracy to predict the category present in the remaining subset of trials (test dataset). This procedure was repeated five times, using a different split for the training and testing datasets. The average classifier’s performance across all repetitions was considered to be the decoding accuracy of the classifier. Because the classification was binary, and the two alternatives were equally probable, the chance performance was 0.5. A bootstrap procedure was performed to estimate the statistical significance of the low-resolution neuronal population decoding. The procedure splits the decoding feature vector into two datasets, training dataset (80% trials) and test dataset (20% trials). From the training set, we constructed a new dataset by randomly resampling the same number of trials as the dataset with replacement, trained a classifier with 5-fold cross validation on that dataset, and tested the classifier’s performance on the test dataset. This procedure was repeated 1,000 times to create a bootstrap distribution of decoding accuracy, and its median and 95% confidence interval (CI) were estimated. The proportion of values in that distribution that was smaller than the chance level was calculated as the *p* value for the null hypothesis that decoding accuracy does not differ from the chance level. Corrections for multiple comparisons were performed using Benjamini and Hochberg’s false discovery rate method (Benjamini and Hochberg, 1995). We set our threshold for significance across all tests at *p* < 0.05.

**Figure 3:**
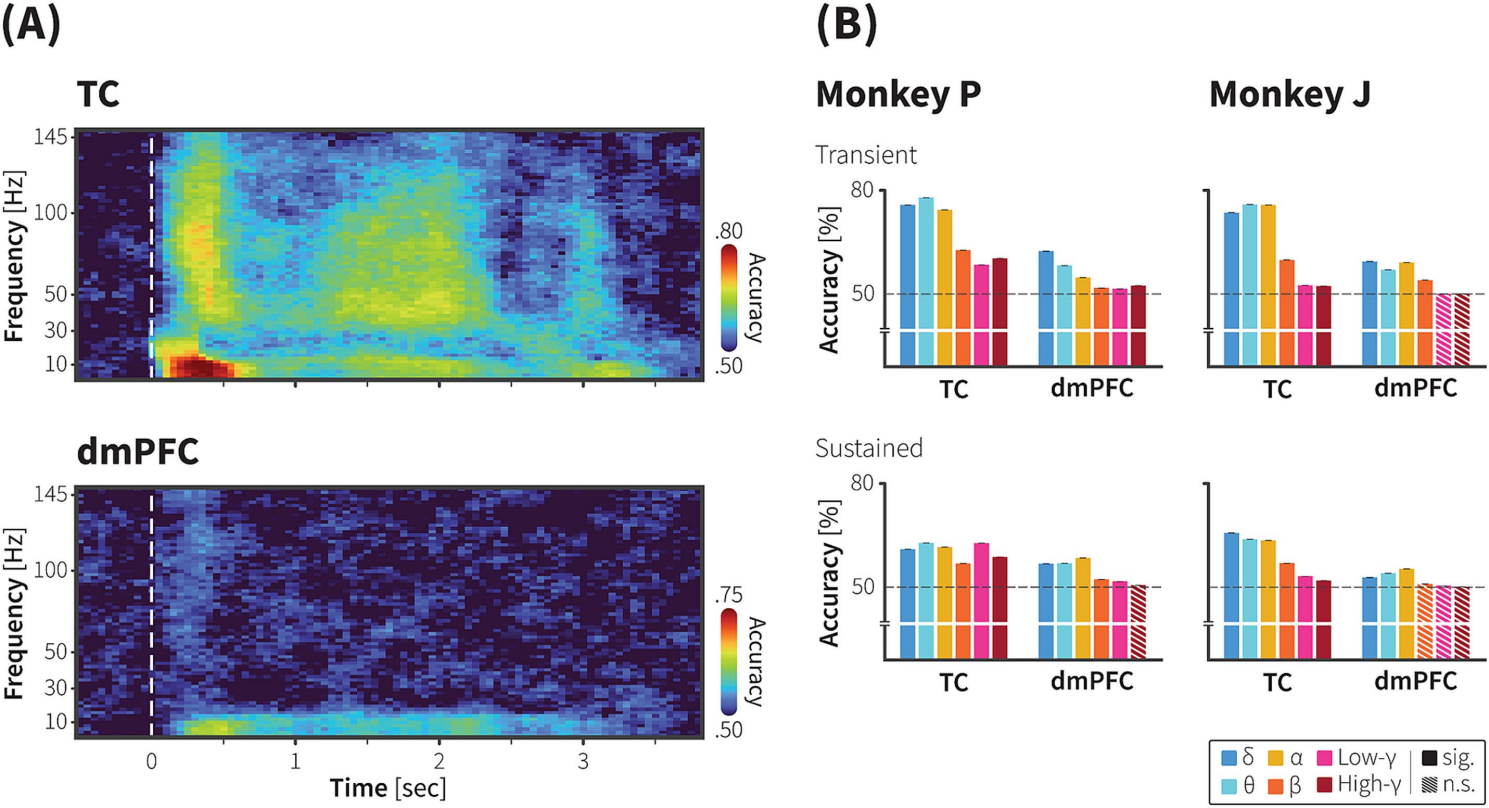
Decoding of animate/inanimate category from population neural activity in the TC and dmPFC. **(A)** Representative time course of animacy using ECoG signals recorded from the TC (upper) and dmPFC (lower) of monkey P. A linear SVM with 5-fold cross validation was trained to decode category information using the power spectra of the high-resolution ERSP as input features (see methods for further details). In monkey P’s dataset, there were 538 stimuli across 1,667 trials (271 animate stimuli across 838 trials). Dashed line indicates onset of the movie stimuli. Color scale bars indicate decoding accuracy. **(B)** Decoding accuracy with transient (upper) and sustained (lower) components of frequency-specific ECoG powers in the TC and dmPFC of monkey P (left) and monkey J (right). The median decoding accuracies obtained from 1,000 iterations were plotted. Error bars indicate 95% CI of bootstrap distribution. Dashed lines indicate the chance level. Colors indicate frequency bands. See Method for details.

To compute frequency-wise decoding in the population activity (Figure 3B), first, we decomposed LFP using the multitaper method with a slightly longer window (500 ms) and step size (350 ms). These parameters yielded time-frequency dimensions with 13 time points and 76 frequency points (frequency resolution of 2 Hz). We calculated the decoding accuracy of each frequency point at two time points, i.e., between −98 – 602 ms and 1,652–2,352 ms, representing transient and sustained activities, respectively. Decoding accuracy for each frequency band were estimated as the median accuracy across frequency points within each frequency band: delta (2–4 Hz), theta (5–8 Hz), alpha (9–13), beta (14–30), low gamma (31–70 Hz), and high gamma (71–200 Hz) (Brovelli et al., 2004; Whittingstall and Logothetis, 2009). We employed the bootstrap procedure to estimate statistical significance and to estimate the variance of the classifiers presented as 95% CI in Figure 3B.

**Figure 4:**
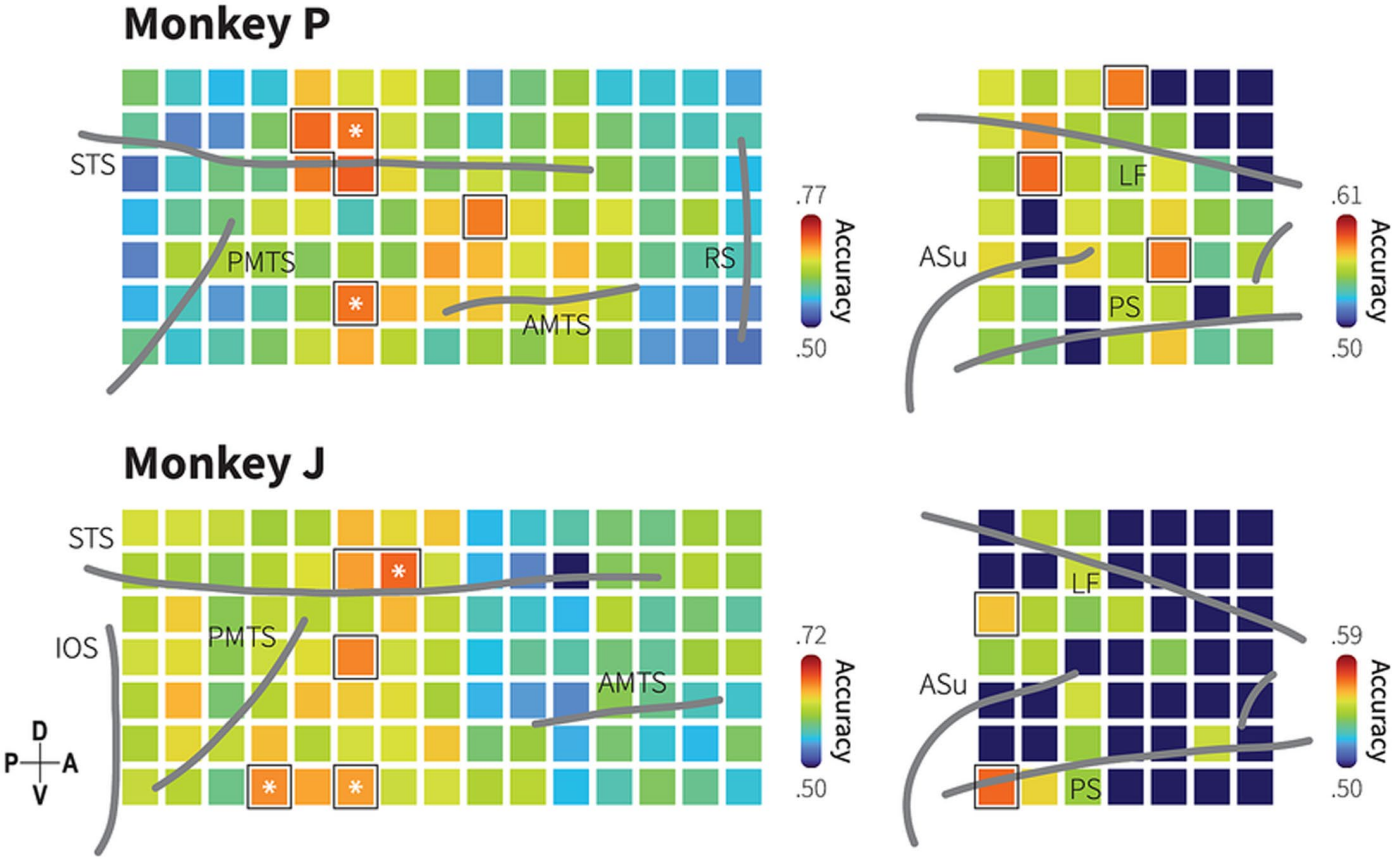
Spatial distribution of category information in the TC and dmPFC. Decoding accuracy maps with searchlight classifiers for animacy information based on LFP in monkeys P (upper) and J (lower). Squares represent ROIs indicating the center of a searchlight area with a 2 × 2 electrode radius. Colors indicate median of searchlight decoding accuracy at each region of interest (ROI) obtained from 1,000 iterations. Dark blue color indicates decoding accuracy not significantly higher than the chance level (bootstrap test; *p* > 0.05; false discovery rate [FDR] correction for 154 ROIs). Black borders indicate the ROIs with decoding performance greater than the 95th percentile of the 105 ROIs in the TC and the 49 ROIs in the dmPFC. Asterisk symbols indicate the informative subregions that show spatial correspondence with the channels to which the most informative IC strongly projects. Grey lines indicate the lip of sulci (similar as previously described in Figure 1C and Supplementary Figure S2).

In searchlight analysis, for each ROI (radius 2.5 mm x 2.5 mm, 2 × 2 electrodes), the feature vector contained LFP data from −100 ms – 2000 ms across all trials resulting in a feature matrix of 1,667 × 4,204 for monkey P and 2,109 × 4,204 for monkey J. We computed the decoding accuracy using a linear SVM with a 5-fold cross-validation and mapped it onto the centre of the ROI. We then repeated this procedure for entire ROI to create a decoding accuracy map, as shown in Figure 4. The statistical significance of the classifier’s performance was tested using the bootstrap procedure.

In the independent component (IC) decoding, IC activities for each epoch were transformed into low- and high-resolution time-frequency data matrices using the multitaper method, as previously described (see ECoG recordings). A linear SVM with 5-fold cross-validation was employed to calculate the decoding accuracy for each time-frequency point. Informative ICs were identified using a permutation procedure in the low-resolution decoding analysis. The procedure involved shuffling the category labels before training the linear SVM. The IC decoding value was defined as the average decoding performance from 300 ms – 2050 ms across six frequencies. We repeated the procedure 1,000 times to create a permutation distribution. Statistical significance was determined by comparing the unshuffled-label decoding accuracy against the permutation distribution. Statistical values were corrected using the Bonferroni test. ICs with adjusted *p* < 0.05 was considered as an informative IC.

### 2.10 Independent component analysis

Prior to applying ICA, segmented LFP data was concatenated to create a matrix of continuous LFP data, $X\left(t\right)=\left\{x_{1}\left(t\right),,..,,x_{i}\left(t\right)\right\}$ , which rows indicate number of channels and columns represent the time course. After centering and whitening, the continuous LFP matrix was inputted as predictors in the CUDAICA-extended Infomax algorithm (Raimondo et al., 2012) to extract independent source signals (or ICs activity), $S\left(t\right)$ . The ICA algorithm assumes that $X\left(t\right)$ is a linear combination of a mixing matrix, M , and $S\left(t\right)$ . Additionally, the number of ICs and the number of channels is assumed to be same, therefore the matrix M must be square and full rank (linearly independent). The output of ICA is an estimated unmixing (weight) matrix, U , where mathematical inverse of the weight matrix, $U^{\left(-1\right)}$ , equals the matrix M. Finally, $S\left(t\right)$ was produced when the matrix U multiplied by $X\left(t\right)$ . By visually inspecting the time-locked activity, ERSP, intertrial coherence of the ICs, we identified a few noise components containing signal artifacts, such as irregular high voltage or trials artifacts, and marked those components for removal.

Based on presumptions underlying ICA decomposition, this procedure also provided us with additional information about the location of the sources. To visualize the location of the ICs activity, the matrix, $U^{\left(-1\right)}$ , was calculated and plotted as topographic maps. This map represents back-projection of single IC onto the electrodes, with values indicating relative contributions of each electrode to the projected IC.

### 2.11 Connectivity analysis

**Figure 5:**
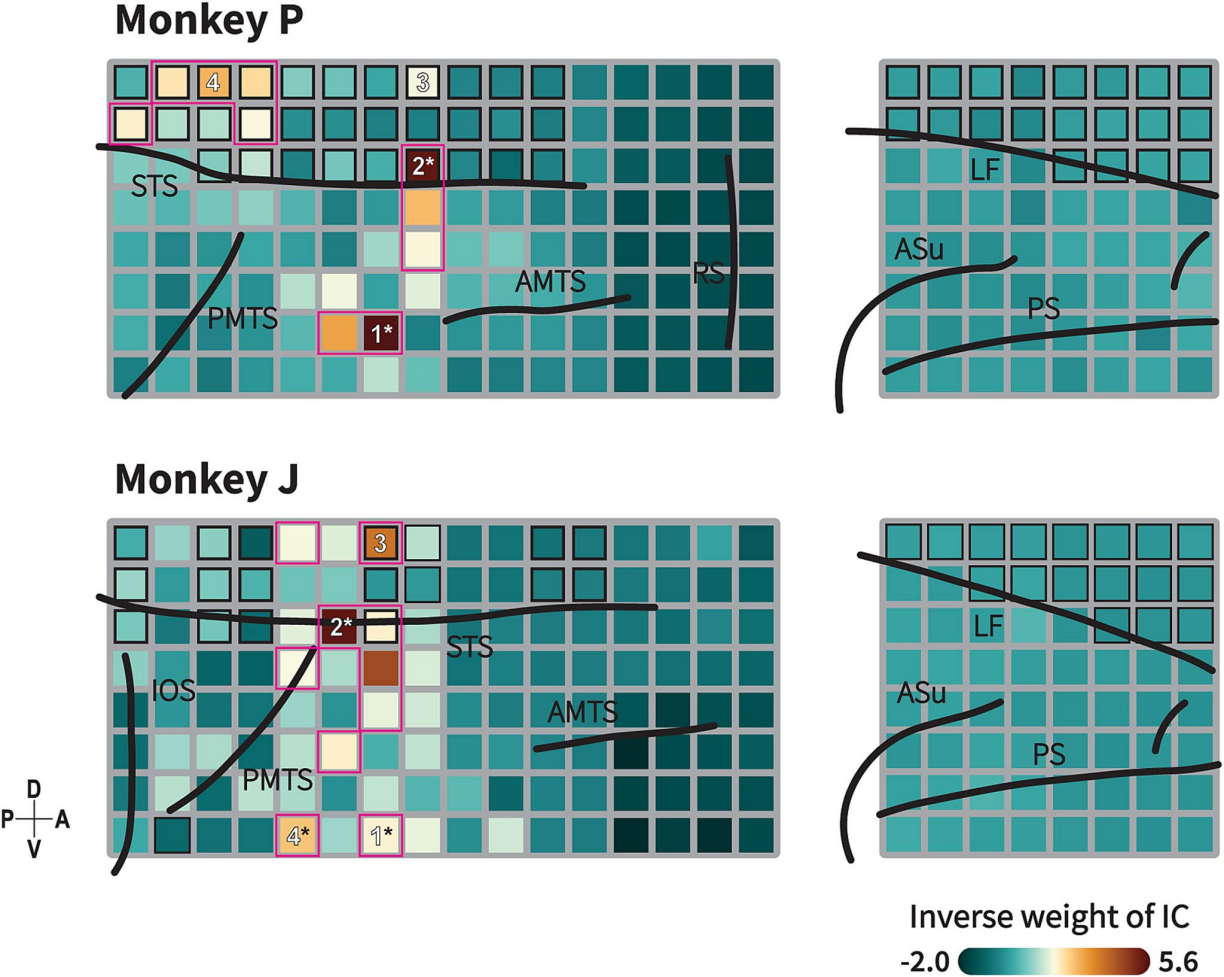
Animate/inanimate category-selective subnetwork identified by Independent Component (IC) Analysis. Topography of ICs with highest decoding performance showing functional subnetwork related to animacy category in monkeys P (upper) and J (lower). This topographical map represents back projection of the IC to each channel. Formats are as in Figure 1C. Black-border squares indicate electrode located within the sulcus/fissure. Channel(s) within the magenta-bordered subregions exhibit the component projection (inverse weight value) exceeding the 95th percentile of 192 channels. Numbers indicate the channels selected for the connectivity analysis. Asterisk symbols indicate channels with strong component projections that exhibit spatial correspondence with the informative subregions in the searchlight maps (see Figure 4). Black lines indicate the lip of sulcus (similar as previously described in Figure 1C and Supplementary Figure S2). Color indicates the absolute value of inverse weight of the IC.

**Figure 6:**
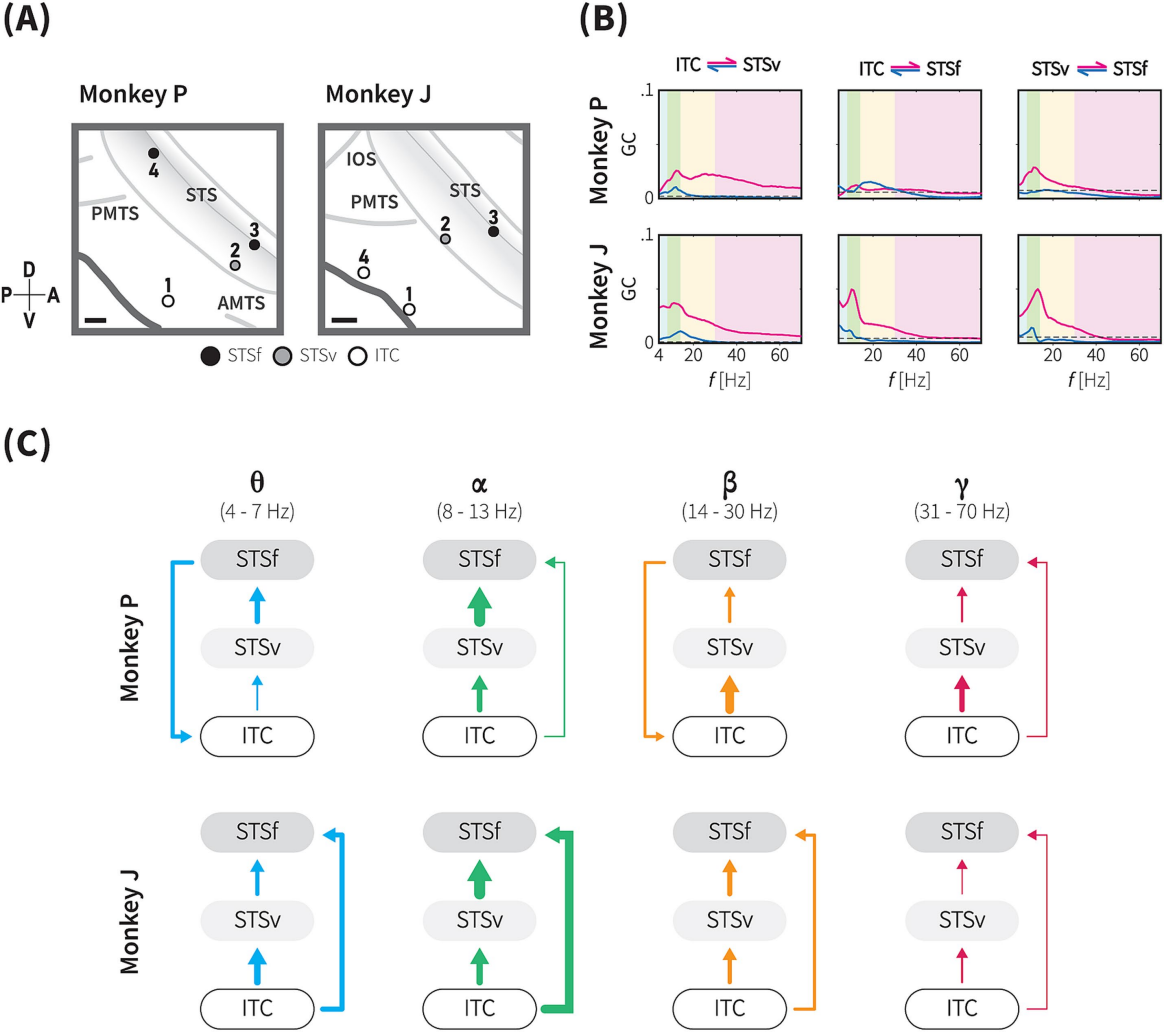
Directional functional connectivity within the animacy network across theta, alpha, beta and gamma frequency bands. **(A)** The positions of the four selected sites for the functional connectivity analysis based on the projection of the informative IC in monkeys P (left) and J (right). See Methods for details. Based on anatomical landmarks, these sites (circles) were classified into three groups, ITC (white), ventral bank of STS (STSv, grey), and fundus of STS (STSf, black). Grey lines indicate the lip and fundus of major sulci. Gray shaded area represents the brain area within the STS. Scale bars, 2 mm. **(B)** Conditional Granger causality (GC) from inter-subregional site-pairs (as indicated by the locations above) in both directions (magenta and blue lines), plotted against frequency (Hz). Blue, green, yellow and magenta shaded areas indicate theta-, alpha-, beta-, and gamma-band frequency ranges, respectively. Dashed lines indicate the significance threshold (1,000-iteration permutation test, *p* < 0.05, with Bonferroni correction for 24 frequency-wise connections). **(C)** Directional GC influence in each frequency band. These diagrams summarize the conditional GC results of monkey P (upper), and J (lower) for each frequency band based on the dGC values. Arrow thickness indicates the dGC value relative to the maximum dGC value across the frequency bands.

Connectivity analysis was performed using the FieldTrip toolbox. Before the analysis, electrodes were selected by locating peak values across the topographical map grid, resulting in the selection of four electrodes (indicated by numbers in Figure 5). To eliminate noise contamination from common recording references, we then performed bipolar derivation to the selected electrodes by taking the difference with their immediately adjacent electrodes on the same lane of the ECoG grids (vertical bipolar derivation). For the selected electrodes located at the dorsal end of a lane, bipolar signals were computed by subtracting the signal of the immediately ventral electrodes. In this analysis, we computed power spectra from our time-series data using the multitapered Fourier transform. Data epochs ranging from 0 ms to 1,000 ms were multitapered using 15 Slepian tapers and then power spectra were computed based on fast Fourier transformation. We specified frequencies of interest in the range from 0 to 150 Hz. We then used the power spectra for calculating conditional nonparametric GC influences. The conditional Granger causality measure causality between two time series, $A\left(t\right)$ and $B\left(t\right)$ , conditional on a third (or more) time series, $C\left(t\right)$ . Suppose that a pairwise GC analysis reveals a causal influence from $A\left(t\right)$ to $B\left(t\right)$ . Conditional Granger causality determines whether this influence has a direct causality or is mediated by $C\left(t\right)$ . For the computation of the conditional GCs, we used multivariate nonparametric spectral matrix factorization (mNPSF). The input to the mNPSF algorithm consisted of the cross-spectral density matrix obtained from the Fourier transformation (Dhamala et al., 2008; Bastos et al., 2015). In both monkeys, pairing four sites formed five inter-subregional pairs and one intra-subregional pair (monkey P: a pair or sites 3 and 4; monkey J: a pair of sites 1 and 4). We then averaged GC influence spectra over pairs including STSf sites (site 3 and 4) in monkey P and ITC sites (site 1 and 4) in monkey J, resulting in six inter-subregional pairs (Figure 6B). GCs values were tested for significance by using a permutation procedure (Brovelli et al., 2004). In the permutation procedure, we created an artificial dataset in which epoch index for each site was randomly permuted. GC spectra were calculated and the largest value along with their corresponding were taken. We repeated this procedure one thousand times to yield the null hypothesis distribution of GC spectra for each pair time series. Then, we compared actual GC obtained from the actual dataset against the 99th percentile value of the null hypothesis to estimate statistical significance. Statistical values were corrected using the Bonferroni test. To examine the directionality of GC influence, we evaluated the differences in GC influence spectra (dGC) across each frequency band, from the theta, alpha, beta, and gamma bands. For each significant GC within these bands, we subtracted the GC value in the opposite direction from the dominant direction. When GC exhibited peaks, we calculated the difference at the peak frequency in the dominant direction. In cases where no peaks were present, we quantified the difference by averaging values across the frequency range. If the opposite direction showed no significant influence, we used the GC value from the significant direction.

## 3 Results

### 3.1 A symbolic animacy categorization task and performance

We trained two Japanese macaques (monkey P and monkey J) to perform a symbolic animate/inanimate categorization task where 2-s natural movie clips depicting the movement of either living or non-living objects were presented (Figures 1A,B). Each movie clip appeared after the monkeys maintained their gaze on a fixation point at the centre of the monitor for 500 ms. Following the movie stimuli, two symbols appeared randomly at different locations on the screen during each trial. The monkeys then had to select the symbol representing the category of the movie clip they had just watched by touching a touchscreen, earning a fluid reward for correct choices. Both monkeys successfully learned the symbolic animate/inanimate task and performed significantly above chance levels before the recording sessions began (monkey P: 98.8%, monkey J: 89.6%), with high recall and precision rates (monkey P: 99.2 and 98.2%; monkey J: 92.0 and 86.7%, respectively).

In the categorical generalization test, the monkeys demonstrated the ability to transfer their learning to new stimuli, showing high discrimination performance even when encountering the stimuli for the first time (monkey P: 83%, monkey J: 67%; one-tailed exact binomial test, *p* < 0.01 and *p* = 0.04, respectively, Supplementary Figure S1A). Additionally, as illustrated in Supplementary Figure S1B, their performance remained consistently high (~82% correct; one-tailed exact binomial test, *p* < 0.01) during trial-unique tests, which included a dataset with a large number of novel movie stimuli (168–300 clips per session) (Methods). These results confirm that the monkeys effectively classified different stimuli based on their animacy features. The reaction time (RT) measurements indicated both monkeys chose the symbol faster for inanimate movies (median RT, monkey P: 904 ms, monkey J 896 ms) compared to animate movies (monkey P: 916 ms; monkey J: 942 ms). The median difference in reaction times was 12 ms for monkey P and 46 ms for monkey J (*p* < 0.001, Mann–Whitney U test; Supplementary Figure S1C).

### 3.2 ECoG responses to animate and inanimate movies in the TC and dmPFC

While the monkeys performed the tasks, we recorded surface LFPs using a 128-channel and 64-channel ECoG electrode arrays implanted subdurally over the monkeys’ right TC and dmPFC, respectively, with a number of channels inserted in the STS (monkey P: 31 channels, monkey J: 18 channels) or longitudinal fissure (monkey P: 20 channels, monkey J: 17 channels; Figure 1C and Supplementary Figure S2). Both areas exhibited visually evoked potential responses to movie stimuli during the task. An event-related potential (ERP) characterized by a multiphasic waveform was strongly elicited by animate/inanimate stimuli upon initial presentation (~600 ms), followed by weaker responses returning to baseline level. ERP components then reappeared during symbol selection (Figure 2, upper).

Time-frequency representation revealed selective time course of mean log power changes from pre-stimulus baseline in response to animate\inanimate stimuli in the TC and dmPFC. As depicted in Figure 2 (middle and lower), in the ITC, animate stimuli elicited greater average power in low (2–16 Hz) and high (30–148 Hz) frequency bands during the initial presentation, followed by a sustained increase in the high frequency bands and sustained suppression in the low frequency bands. The most pronounced increases were observed in the high gamma frequencies during the initial presentation and in the sustained activities.

In the dmPFC, we observed greater power in the low frequency band during the inanimate stimuli presentation. A concurrent but weaker high frequency power was observed during early stimuli presentation. In subsequent sections, we analyzed responses during the movie presentation period to exclude direct effects of motor and reward-related events.

### 3.3 Population activity patterns efficiently decoded the category

We further attempted to evaluate the statistical significance of the category selectivity of neuronal activity at population level. A support vector machine (SVM) classifier was trained to decode category from the time-frequency representation of the neuronal population (Methods). A substantial amount of category information was decoded in both areas. Category information was carried in the low and high frequency band powers, yet each area exhibited different patterns. Category information in the TC was carried by the transition the low frequency band to high frequency band, while category information remained in the low frequency bands in the dmPFC.

Figure 3A shows representative high-resolution time-frequency plots showing the accuracy levels of decoding animacy category from ECoG signals recorded from multiple electrodes in the TC and dmPFC of monkey P. Peaks in decoding performance were observed between 0–600 ms (transient period) and 1,500–2,000 ms (sustained period). Statistical tests were then performed for time-frequency decoding within these peak intervals (300 ms each) as depicted in Figure 3B, demonstrated significance category information decoded with frequency bands at the transient and sustained performance (Methods).

In the TC, we found both frequency bands contained category information above the chance level throughout the stimulus period (Supplementary Figure S3, bootstrap test, *p* < 0.05 with FDR correction for multiple comparison). During the initial presentation of movie, when time-locked response occurred, highest decoding performance was observed with the low frequency band (theta) signals (Figure 3B, 0.81, 95% CI ± 0.07 × 10^−2^; bootstrap test, adjusted *p* < 0.05). However, during the sustained response period, the highest category classification rate (0.63, 95% CI ± 0.03 × 10^−2^, bootstrap test, adjusted *p* < 0.05) was decoded with the high frequency band (low gamma) signals. This indicates a transition of category information from the low frequency band during the transient phase to the high frequency band during the sustained phase in the TC. Results from monkey J, as shown in Supplementary Figure S4, shared similar tendencies with those from monkey P. Category information was decoded above the chance level with both high and low frequency signals in the TC throughout the stimulus period (Supplementary Figure S3, bootstrap test, *p* < 0.05 with FDR correction for multiple comparison). Although the performance with low frequency band signals was relatively higher during the sustained period, decoding accuracy with the high frequency band signals (low gamma) was also significant (0.53, 95% CI ± 0.03 × 10^−2^, bootstrap test, adjusted *p* < 0.05), which peaked just before the stimulus end, nearly at the same time as in monkey P.

For the dmPFC, the high-resolution time-frequency plot indicated that category information was mainly decoded from the low frequency band signals. Decoding performance with the high frequency band signals was slightly above the chance level in the monkey P, but for monkey J, it did not exceed the chance level as shown in Figure 3B. Thus, decoding accuracy with the low frequency band signals in the dmPFC remained significant throughout the stimulus period across the two monkeys.

### 3.4 Spatial distribution of category information revealed by searchlight analysis

Given the selectivity in both areas, we conducted the searchlight analysis to identify spatial specificity in encoding animacy categories (Figure 4). While a majority of local region of interest (ROI) covered by 2 × 2 electrodes, each of which covering a 2.5 mm by 2.5 mm area (Methods), exhibited selectivity for animacy category with decoding performance above the chance level (monkey P: 105 ROIs in the TC and 38 ROIs in the dmPFC, monkey J: 104 ROIs in the TC and 16 ROIs in the dmPFC; evaluated at *p* < 0.05, bootstrap test with FDR correction for the number of the ROIs), the ROI with the highest decoding accuracy in the TC was located in the ventral bank of the STS, anterodorsal to the posterior middle temporal sulcus (PMTS). Additionally, high category selectivity was observed in regions anterior to the PMTS and dorsal to the anterior middle temporal sulcus (AMTS).

Searchlight analysis revealed that category information was distributed throughout the dmPFC with relatively lower decoding performances compared to the TC. Although the highest decoding performance was observed in different locations between the monkeys, dorsoposterior dmPFC for monkey P and the caudal part of the principal sulcus for monkey J, both monkeys exhibited significant category selectivity in these locations. These results suggest that the category information is encoded in distributed areas within the TC and dmPFC, in which interconnectivity between focal ROIs might reflect possible functional architecture for animacy categorization. Do these distributed pieces of information indicate independent parallel pathways of information processing, or do they suggest interdependent and cooperative pathways? We subsequently investigated this question.

### 3.5 Identification of distributed functional networks using independent component analysis in the TC and dmPFC

ICA is a well-known approach for identifying functionally connected networks from signals recorded by fMRI (McKeown et al., 1998; Moeller et al., 2009) or LFP signals (Orellana et al., 2024). We applied ICA to decompose our LFP signals into 192 statistically independent source signals, along with their spatial distribution maps. Time-frequency decoding analysis were then conducted to identify informative component for the category discrimination (Methods). The decoding analysis revealed that a subset of independent components (IC) was informative for animate/inanimate categorization with high decoding performance observed in the high and low frequency bands (Supplementary Figure S5; monkey P: 101 ICs, monkey J: 28 ICs; *p* < 0.05, the permutation test with FDR correction for 192 ICs).

A topographical map of the most informative component is shown in Figure 5. The map depicts the IC projected across multiple regions within the TC, indicating its cooperative activity in these regions. In monkey P, the component exhibited strong projections to electrodes in the ventral bank of the STS and in a gyral part of the TC posterior to the AMTS. Additionally, the component also projected to electrodes in the fundus of the STS dorsal to the PMTS. In monkey J, strong projections were observed in the ventral bank of the STS, a gyral region dorsal to the PMTS, with additional projections in distributed regions anterior to the PMTS and in the fundus of the STS.

We observed that the subregions to which the informative IC strongly projected––specifically, the ventral bank of the STS and the gyral part of the TC––spatially corresponded with the ROIs exhibiting high decoding performance in the TC (as indicated by asterisk in Figures 4, 5). Previous studies have demonstrated that motion processing is coordinated in the STS region (Oram and Perrett, 1994, 1996; Nelissen et al., 2006; Vangeneugden et al., 2011; Jastorff et al., 2012), while shape processing is coordinated in the gyrus part of the TC (Tanaka, 1996; Desimone et al., 1984; Tsao, 2014). Our results suggest the existence of an integrated subnetwork combining these circuits associated with animacy categorization. Furthermore, we investigated the direction of the functional connectivity within this integrated subnetwork.

### 3.6 Directed functional connectivity within the animacy subnetwork

Based on the above findings, where a functional subnetwork was identified through the cortical projection of the most informative component (Figure 5), we further evaluated conditional GC influences to determine the presence and direction of category information flow within this network. Our analysis focused on four locations exhibiting strong projections from the most informative IC (Figures 5, 6A), which were grouped into three main regions: the gyrus of the TC (ITC), the ventral bank of the STS (STSv), and the fundus of the STS (STSf). GC values were calculated using non-parametric methods, with bidirectional conditional GC spectra computed for all pairwise combinations (Figure 6B). To assess the directionality of the significant connections, we calculated the difference in GC influences (dGC) for each frequency band.

We identified significant frequency-specific directional influences among the ITC, STSv, and STSf, indicating functional connectivity within the animacy-responsible network (20 out of 24 frequency-wise connections for P, 21 out of 24 frequency-wise connections for J, Figure 6B and Table 1). Unidirectional significant flow and the dGC analysis revealed a general directional functional flow from the ITC to the STS in both monkeys (Figure 6C; Supplementary Figure S6). Consistently across both monkeys, signalling in the subnetwork was prominently unidirectional from the ITC to STS (ITC → STSv, STSv → STSf, and ITC → STSf) in the alpha and gamma bands, as well as in the theta and beta bands (ITC → STSv, STSv → STSf) (Figure 6C). GC influence spectra in 4 out of 6 pairs peaked within the alpha band range forming unidirectional connections from the ITC to STS, while substantial amounts of GC peaks within theta band range were observed from the STSf to ITC (Figure 6B).

**Table 1:** Conditional GC values between sub-regions in Monkeys P and J.

			TO		
	Subregion		ITC	STSv	STSf
	Monkey P				
FROM	ITC	ϒ	–	0.0141	0.0062
		β	–	0.0227*	0.0078
		α	–	0.0257*	0.0124*
		θ	–	0.0099	0.0029
	STSv	ϒ	n.s.	–	0.0057
		β	0.0019	–	0.0152
		α	0.0106	–	0.0287*
		θ	0.0049	–	0.0152
	STSf	ϒ	0.0025	n.s.	–
		β	0.0153*	0.0066	–
		α	0.0084	n.s.	–
		θ	0.0116*	n.s.	–
	Monkey J				
FROM	ITC	ϒ	–	0.0094	0.0061
		β	–	0.0234	0.0168
		α	–	0.0372*	0.0497*
		θ	–	0.0343*	0.0397*
	STSv	ϒ	0.0004	–	0.0051
		β	0.0047	–	0.0227
		α	0.0081	–	0.0500*
		θ	0.0046	–	0.0266
	STSf	ϒ	n.s.	n.s.	–
		β	0.0027	n.s.	–
		α	0.0107	0.0025	–
		θ	0.0168	0.0076	–

Direction of influence is from sites listed on the left to the site at the top. Unless otherwise indicated, averaged GC values for each frequency band are displayed in separate rows. Values are shown only for frequency bands with significant GC spectra (see Methods). ‘n.s.’ denotes not significant; ‘–’ indicates a self-pairing site. ‘*’ marks the peak of GC spectra in each pair. ITC, inferior temporal cortex; STSv, ventral bank of STS; STSf, fundus of STS.

In monkey P, all six inter-subregional pairs showed significant bidirectional causal influences, confirming a functional network integrating the gyrus and the sulcus parts of the TC (adjusted *p* < 0.05, permutation test with Bonferroni correction; Table 1). GC spectra analysis in the pairs of the ITC and both STS subregions exhibited significant bidirectional influences in the theta, alpha, and beta bands, while bidirectional influences in the gamma band were only observed in the ITC – STSf pair (Supplementary Figure S6, upper panel). Additionally, between the STS subregions, we observed the STSv → STSf direction consistently had significant influences across the frequency bands, whereas significant GC values in the reverse direction were observed solely in the beta band.

In monkey J, all inter-subregional pairs exhibited significant causal influences in both directions (Table 1). Generally, the GC spectra demonstrated a pattern resembling the subnetwork characteristics observed in monkey P. For instance, pairs of the ITC and both STS subregions had significant bidirectional influences in theta, alpha, and beta band (Supplementary Figure S6, lower panel). In consistent with monkey P, results of the monkey J showed unidirectional connections in alpha and gamma band, which suggests a hierarchical organization within the animacy subnetwork (Figure 6C and see Discussion). Unlike the monkey P, the GC influence spectra in the theta and beta bands were stronger in the ITC → STSf direction than in the reverse direction, indicating unidirectional signalling from the ITC to STS.

## 4 Discussion

Introducing a symbolic animate/inanimate categorization task using natural movie clips, we showed that monkeys achieved high performance levels and demonstrated robust generalization to novel stimuli. Concurrently, the wide areal ECoG recordings from the TC and dmPFC revealed sufficient information for animacy categorization not only at the onset of the movie but throughout the entire presentation period in both areas. Spatial analysis highlighted specific regions within the TC and dmPFC, particularly in the ITC (inferior gyrus of the temporal cortex), the ventral bank of the STS, and the fundus of the STS, which exhibited high category selectivity. ICA analysis identified functionally connected networks across these regions, suggesting integrated pathways for animacy categorization. Granger causality analysis confirmed the functional connectivity and further indicated directional information flow, emphasizing the pronounced alpha band influences within the identified network. These findings suggest a distributed but functionally interconnected neural substrate for animacy processing across the TC and underscore the subnetwork integrating the gyrus (ITC) and the sulcus parts of the TC (STS) involved in natural animacy categorization in macaque monkeys. The results were comparable to previous ECoG studies in humans (Matsuo et al., 2015; Rupp et al., 2017; Rogers et al., 2021) which have shown that animacy information is encoded in the VTC, potentially corresponding functionally to the ITC and STS in monkeys.

Animacy recognition in monkeys has been studied using various tasks, such as gaze measurement and go/no-go tasks in the past (Fabre-Thorpe et al., 1998; Tsutsumi et al., 2012; Yetter et al., 2021). Unlike the go/no-go tasks, where a specific behavior must be associated with a specific category (animate/inanimate), using explicit symbols as the response targets for monkeys allowed for an unbiased investigation of neural responses related to animacy recognition. This task paradigm was better suited for evaluating sustained activities occurring around the response time for the monkeys. We observed clear differences in the frequency representation of sustained activity between the TC and dmPFC. In the TC, category information was observed in both high and low frequencies, while in the dmPFC, it was primarily observed in low frequencies. This suggests that information related to the same cognitive process might be represented in different frequency bands depending on the stage of processing.

The regions identified as informative in this study, particularly the ventral bank and fundus of the STS, are consistent with previous findings that suggest the involvement of STS cells in processing biological motion (Perrett et al., 1989; Giese and Poggio, 2003; Nelissen et al., 2006; Vangeneugden et al., 2011; Jastorff et al., 2012). Traditionally, the STS has been viewed as a convergence point for the dorsal and ventral pathways, which are responsible for processing motion and shape information, respectively, based on anatomical location and fiber connections (Bruce et al., 1981; Seltzer and Pandya, 1978, 1991; Farivar, 2009). In this study, however, informative regions were also identified in the gyral part of the TC. This may be attributed to the rich shape attributes in the natural movies we used, in addition to the rich motion attributes. Importantly, as revealed by ICA and Granger causality analyses, the informative regions inside of the sulcus (the STS) and outside of it (ITC) demonstrated strong functional connectivity. This finding suggests that the distributed regions are not merely representing information related to motion and shape separately but are forming a subnetwork that collectively represents animacy information.

Recent research also suggests the presence of face and body patches within the STS, which process face and body movements exclusively and independently from the dorsal visual pathway (Hesse and Tsao, 2020; Yang and Freiwald, 2023). Furthermore, another study contrasting responses to animate and inanimate stimuli using face and body representations demonstrated a nuanced interaction between face and body processing in animacy perception (Bao et al., 2020). Although some of the animacy-related movies in this study included faces and bodies, the relationship between these patches and the observed activity remains unclear due to the lack of controlled stimuli specifically targeting these patches. Additionally, while our multi-channel ECoG grid is able to measure LFP from both surface and intrasulcal areas in the ITC, it is difficult to distinguish whether the recordings were obtained from the ventral or dorsal bank of the sulcus, making it challenging to clarify the exact correspondence of these regions (Freiwald et al., 2016).

In visual areas, it is known that the frequency bands involved in Granger causality and hierarchical processing follow a pattern in which feedforward information is carried in the theta and gamma bands, while feedback information is transmitted in the alpha and beta bands (Van Kerkoerle et al., 2014; Bastos et al., 2015). In the present study, our results suggest that signals propagate from the ITC to the STS in both feedforward (theta and gamma bands) and feedback (alpha and beta bands) representations. However, given that the GC peaks often occur in the alpha band, these connections may lean more toward feedback processing. This pattern may reflect a methodological difference from previous studies that focused only on peak frequencies, while our study considered the full range of frequency bands. It may also reflect the fact that the network we studied is confined to adjacent regions. The feedback pathway from shape to motion may share mechanisms with processes involved in detecting motion from static snapshots (Singer and Sheinberg, 2010). In contrast, for feedforward pathways, the connection from the STSf to ITC exhibits the second-highest peak in monkey P, a point of interest shared across both monkeys. The feedforward pathway from motion to shape in animacy recognition underscores the importance of constructing shape information from motion, potentially sharing mechanisms with biological dot motion perception (Oram and Perrett, 1994; Giese and Poggio, 2003) and the detection of shape from motion when no distinct shape is present (Burk and Sheinberg, 2022). Further research with stimuli that systematically manipulate form and motion dimensions is needed to explore these neuronal dynamics. While GC analysis revealed the direction of information flow within the subnetworks, the specific content of the information underlying these functional connections remains an open question for future research.

In this study, the dmPFC also exhibited significant category information, in line with previous findings (Mitchell et al., 2002). Although the most informative components did not peak in the dmPFC (Figure 5), some lower-tier informative components did, and others showed peaks in both regions (data not shown). Considering that general category responses are observed in more lateral regions of the PFC in monkeys (Freedman et al., 2002; Shima et al., 2007; Roy et al., 2010), expanding the recording areas in future studies will also be important.

Animacy perception involves a cognitive process of determining whether an object is alive or dead, or whether it possesses emotions or intentions (Rakison and Poulin-Dubois, 2001; Barrett and Behne, 2005). Such perception can be considered a part of conceptual cognition, as it involves recognizing abstract concepts and intentions. In other words, animacy perception goes beyond simple sensory information processing and includes elements that facilitate a conceptual understanding of objects and environments. This study’s results demonstrate the importance of the temporal cortical functional network in animacy visual processing when an individual is viewing natural scenes. By further identifying the processing between the TC and PFC regions, we can enhance our understanding of the process of abstraction in animacy recognition.

## Data Availability

The original contributions presented in the study are included in the article/Supplementary material, further inquiries can be directed to the corresponding author.

## Footnotes

^1^ https://zulko.github.io/moviepy/

## References

[ref1] Amodio D. M. Frith C. D. (2006). Meeting of minds: the medial frontal cortex and social cognition. Nat. Rev. Neurosci. 7, 268–277. doi: 10.1038/nrn1884, PMID: 16552413

[ref2] Bao P. She L. McGill M. Tsao D. Y. (2020). A map of object space in primate inferotemporal cortex. Nature 583, 103–108. doi: 10.1038/s41586-020-2350-5, PMID: 32494012 PMC8088388

[ref3] Barrett H. C. Behne T. (2005). Children’s understanding of death as the cessation of agency: a test using sleep versus death. Cognition 96, 93–108. doi: 10.1016/j.cognition.2004.05.004, PMID: 15925571

[ref4] Bastos A. M. Vezoli J. Bosman C. A. Schoffelen J. M. Oostenveld R. Dowdall J. R.. (2015). Visual areas exert feedforward and feedback influences through distinct frequency channels. Neuron 85, 390–401. doi: 10.1016/j.neuron.2014.12.018, PMID: 25556836

[ref5] Bell A. H. Hadj-Bouziane F. Frihauf J. B. Tootell R. B. H. Ungerleider L. G. (2009). Object representations in the temporal cortex of monkeys and humans as revealed by functional magnetic resonance imaging. J. Neurophysiol. 101, 688–700. doi: 10.1152/jn.90657.2008, PMID: 19052111 PMC2657058

[ref6] Benjamini Y. Hochberg Y. (1995). Controlling the false discovery rate: a practical and powerful approach to multiple testing. J. R. Statist. Soc. B 57, 289–300. doi: 10.1111/j.2517-6161.1995.tb02031.x

[ref7] Bokil H. Andrews P. Kulkarni J. E. Mehta S. Mitra P. P. (2010). Chronux: a platform for analyzing neural signals. J. Neurosci. Methods 192, 146–151. doi: 10.1016/j.jneumeth.2010.06.020, PMID: 20637804 PMC2934871

[ref8] Brovelli A. Ding M. Ledberg A. Chen Y. Nakamura R. Bressler S. L. (2004). Beta oscillations in a large-scale sensorimotor cortical network: directional influences revealed by granger causality. PNAS 101, 9849–9854. doi: 10.1073/pnas.0308538101, PMID: 15210971 PMC470781

[ref9] Bruce C. Desimone R. Gross C. G. (1981). Visual properties of neurons in a polysensory area in superior temporal sulcus of the macaque. J. Neurophysiol. 46, 369–384. doi: 10.1152/jn.1981.46.2.369, PMID: 6267219

[ref10] Burk D. C. Sheinberg D. L. (2022). Neurons in inferior temporal cortex are sensitive to motion trajectory during degraded object recognition. Cereb Cortex Commun. 3:tgac034. doi: 10.1093/texcom/tgac034, PMID: 36168516 PMC9499820

[ref11] Chao L. L. Haxby J. V. Martin A. (1999). Attribute-based neural substrates in temporal cortex for perceiving and knowing about objects. Nat. Neurosci. 2, 913–919. doi: 10.1038/13217, PMID: 10491613

[ref12] de Cheveigné A. (2020). ZapLine: a simple and effective method to remove power line artifacts. NeuroImage 207:116356. doi: 10.1016/j.neuroimage.2019.116356, PMID: 31786167

[ref13] Delorme A. Makeig S. (2004). EEGLAB: an open source toolbox for analysis of single-trial EEG dynamics including independent component analysis. J. Neurosci. Methods 134, 9–21. doi: 10.1016/j.jneumeth.2003.10.009, PMID: 15102499

[ref14] Desimone R. Albright T. D. Gross C. G. Bruce C. (1984). Stimulus-selective properties of inferior temporal neurons in the macaque. J. Neurosci. 4, 2051–2062. doi: 10.1523/JNEUROSCI.04-08-02051.1984, PMID: 6470767 PMC6564959

[ref15] Dhamala M. Rangarajan G. Ding M. (2008). Analyzing information flow in brain networks with nonparametric granger causality. NeuroImage 41, 354–362. doi: 10.1016/j.neuroimage.2008.02.020, PMID: 18394927 PMC2685256

[ref16] Fabre-Thorpe M. Richard G. Thorpe S. J. (1998). Rapid categorization of natural images by rhesus monkeys. Neuroreport 9, 303–308. doi: 10.1097/00001756-199801260-00023, PMID: 9507973

[ref17] Farivar R. (2009). Dorsal-ventral integration in object recognition. Brain Res. Rev. 61, 144–153. doi: 10.1016/j.brainresrev.2009.05.006, PMID: 19481571

[ref18] Freedman D. J. Riesenhuber M. Poggio T. Miller E. K. (2002). Visual categorization and the primate prefrontal cortex: neurophysiology and behavior. J. Neurophysiol. 88, 929–941. doi: 10.1152/jn.2002.88.2.929, PMID: 12163542

[ref19] Freiwald W. Duchaine B. Yovel G. (2016). Face processing systems: from neurons to real-world social perception. Annu. Rev. Neurosci. 39, 325–346. doi: 10.1146/annurev-neuro-070815-013934, PMID: 27442071 PMC5345271

[ref20] Giese M. A. Poggio T. (2003). Cognitive neuroscience: neural mechanisms for the recognition of biological movements. Nat. Rev. Neurosci. 4, 179–192. doi: 10.1038/nrn1057 12612631

[ref21] Grandchamp R. Delorme A. (2011). Single-trial normalization for event-related spectral decomposition reduces sensitivity to noisy trials. Front. Psychol. 2:236. doi: 10.3389/fpsyg.2011.00236, PMID: 21994498 PMC3183439

[ref22] Hauser M. D. (1998). A nonhuman primate’s expectations about object motion and destination: the importance of self-propelled movement and animacy. Dev. Sci. 1, 31–37. doi: 10.1111/1467-7687.00009

[ref23] Hesse J. K. Tsao D. Y. (2020). The macaque face patch system: a turtle’s underbelly for the brain. Nat. Rev. Neurosci. 21, 695–716. doi: 10.1038/s41583-020-00393-w, PMID: 33144718

[ref24] Jastorff J. Popivanov I. D. Vogels R. Vanduffel W. Orban G. A. (2012). Integration of shape and motion cues in biological motion processing in the monkey STS. NeuroImage 60, 911–921. doi: 10.1016/j.neuroimage.2011.12.087, PMID: 22245356

[ref25] Kaiju T. Doi K. Yokota M. Watanabe K. Inoue M. Ando H.. (2017). High spatiotemporal resolution ECoG recording of somatosensory evoked potentials with flexible micro-electrode arrays. Front. Neural. Circuits 11:20. doi: 10.3389/fncir.2017.00020, PMID: 28442997 PMC5386975

[ref26] Kiani R. Esteky H. Mirpour K. Tanaka K. (2007). Object category structure in response patterns of neuronal population in monkey inferior temporal cortex. J. Neurophysiol. 97, 4296–4309. doi: 10.1152/jn.00024.2007, PMID: 17428910

[ref27] Klug M. Kloosterman N. A. (2022). Zapline-plus: a Zapline extension for automatic and adaptive removal of frequency-specific noise artifacts in M/EEG. Hum. Brain Mapp. 43, 2743–2758. doi: 10.1002/hbm.25832, PMID: 35278015 PMC9120550

[ref28] Kriegeskorte N. Mur M. Ruff D. A. Kiani R. Bodurka J. Esteky H.. (2008). Matching categorical object representations in inferior temporal cortex of man and monkey. Neuron 60, 1126–1141. doi: 10.1016/j.neuron.2008.10.043, PMID: 19109916 PMC3143574

[ref29] Matsuda K. Nagami T. Sugase Y. Takemura A. Kawano K. (2017). “A widely applicable real-time mono-binocular eye tracking system using a high frame-rate digital camera” in Human-computer interaction. User Interface design, development and multimodality. ed. Kurosu M. (Cham: Springer International Publishing), 593–608.

[ref30] Matsuo T. Kawasaki K. Kawai K. Majima K. Masuda H. Murakami H.. (2015). Alternating zones selective to faces and written words in the human ventral occipitotemporal cortex. Cereb. Cortex 25, 1265–1277. doi: 10.1093/cercor/bht319 24285843

[ref31] Matsuo T. Kawasaki K. Osada T. Sawahata H. Suzuki T. Shibata M.. (2011). Intrasulcal electrocorticography in macaque monkeys with minimally invasive neurosurgical protocols. Front. Syst. Neurosci. 5:34. doi: 10.3389/fnsys.2011.00034, PMID: 21647392 PMC3103840

[ref32] McKeown M. J. Makeig S. Brown G. G. Jung T. P. Kindermann S. S. Bell A. J.. (1998). Analysis of fMRI data by blind separation into independent spatial components. Hum. Brain Mapp. 6, 160–188. doi: 10.1002/(SICI)1097-0193(1998)6:3<160::AID-HBM5>3.0.CO;2-1 9673671 PMC6873377

[ref33] Mitchell J. P. Heatherton T. F. Macrae C. N. Raichle M. E. (2002). Distinct neural systems subserve person and object knowledge. PNAS 99, 15238–15243. doi: 10.1073/pnas.232395699, PMID: 12417766 PMC137574

[ref34] Moeller S. Nallasamy N. Tsao D. Y. Freiwald W. A. (2009). Functional connectivity of the macaque brain across stimulus and arousal states. J. Neurosci. 29, 5897–5909. doi: 10.1523/JNEUROSCI.0220-09.2009, PMID: 19420256 PMC6665231

[ref35] Naselaris T. Stansbury D. E. Gallant J. L. (2012). Cortical representation of animate and inanimate objects in complex natural scenes. J. Physiol. Paris 106, 239–249. doi: 10.1016/j.jphysparis.2012.02.001, PMID: 22472178 PMC3407302

[ref36] Nelissen K. Vanduffel W. Orban G. A. (2006). Charting the lower superior temporal region, a new motion-sensitive region in monkey superior temporal sulcus. J. Neurosci. 26, 5929–5947. doi: 10.1523/JNEUROSCI.0824-06.2006 16738235 PMC6675228

[ref37] Oostenveld R. Fries P. Maris E. Schoffelen J. M. (2011). FieldTrip: open source software for advanced analysis of MEG, EEG, and invasive electrophysiological data. Comput. Intell. Neurosci. 2011, 1–9. doi: 10.1155/2011/156869, PMID: 21253357 PMC3021840

[ref38] Oram M. W. Perrett D. I. (1994). Responses of anterior superior temporal Polysensory (STPa) neurons to “biological motion” stimuli. J. Cogn. Neurosci. 6, 99–116. doi: 10.1162/jocn.1994.6.2.99, PMID: 23962364

[ref39] Oram M. W. Perrett D. I. (1996). Integration of form and motion in the anterior superior temporal Polysensory area (STPa) of the macaque monkey. J. Neurophysiol. 76, 109–129. doi: 10.1152/jn.1996.76.1.109, PMID: 8836213

[ref40] Orellana V. D. Donoghue J. P. Vargas-Irwin C. E. (2024). Low frequency independent components: internal neuromarkers linking cortical LFPs to behavior. iScience 27:108310. doi: 10.1016/j.isci.2023.108310, PMID: 38303697 PMC10831875

[ref41] Perrett D. I. Harries M. H. Bevan R. Thomas S. Benson P. J. Mistlin A. J.. (1989). Frameworks of analysis for the neural representation of animate objects and actions. J. Exp. Biol. 146, 87–113. doi: 10.1242/jeb.146.1.87, PMID: 2689570

[ref42] Pinsk M. A. DeSimone K. Moore T. Gross C. G. Kastner S. (2005). Representations of faces and body parts in macaque temporal cortex: a functional MRI study. PNAS 102, 6996–7001. doi: 10.1073/pnas.0502605102, PMID: 15860578 PMC1100800

[ref43] Raimondo F. Kamienkowski J. E. Sigman M. Fernandez Slezak D. (2012). CUDAICA: GPU optimization of infomax-ICA EEG analysis. Comput. Intell. Neurosci. 2012, 1–8. doi: 10.1155/2012/206972, PMID: 22811699 PMC3395116

[ref44] Rakison D. H. Poulin-Dubois D. (2001). Developmental origin of the animate-inanimate distinction. Psychol. Bull. 127, 209–228. doi: 10.1037/0033-2909.127.2.209, PMID: 11316011

[ref45] Rogers T. T. Cox C. R. Lu Q. Shimotake A. Kikuchi T. Kunieda T.. (2021). Evidence for a deep, distributed and dynamic code for animacy in human ventral anterior temporal cortex. eLife 10:e66276. doi: 10.7554/eLife.66276, PMID: 34704935 PMC8550752

[ref46] Roy J. E. Riesenhuber M. Poggio T. Miller E. K. (2010). Prefrontal cortex activity during flexible categorization. J. Neurosci. 30, 8519–8528. doi: 10.1523/JNEUROSCI.4837-09.2010, PMID: 20573899 PMC3709835

[ref47] Rupp K. Roos M. Milsap G. Caceres C. Ratto C. Chevillet M.. (2017). Semantic attributes are encoded in human electrocorticographic signals during visual object recognition. NeuroImage 148, 318–329. doi: 10.1016/j.neuroimage.2016.12.074, PMID: 28088485

[ref48] Sato T. Uchida G. Lescroart M. Kitazono J. Okada M. Tanifuji M. (2013). Object representation in inferior temporal cortex is organized hierarchically in a mosaic-like structure. J. Neurosci. 33, 16642–16656. doi: 10.1523/JNEUROSCI.5557-12.2013, PMID: 24133267 PMC6618530

[ref49] Seltzer B. Pandya D. N. (1978). Afferent cortical connections and architectonics of the superior temporal sulcus and surrounding cortex in the rhesus monkey. Brain Res. 149, 1–24. doi: 10.1016/0006-8993(78)90584-x, PMID: 418850

[ref50] Seltzer B. Pandya D. N. (1991). Post-rolandic cortical projections of the superior temporal sulcus in the rhesus monkey. J. Comp. Neurol. 312, 625–640. doi: 10.1002/cne.903120412, PMID: 1761745

[ref51] Shima K. Isoda M. Mushiake H. Tanji J. (2007). Categorization of behavioural sequences in the prefrontal cortex. Nature 445, 315–318. doi: 10.1038/nature05470 17183266

[ref52] Singer J. M. Sheinberg D. L. (2010). Temporal cortex neurons encode articulated actions as slow sequences of integrated poses. J. Neurosci. 30, 3133–3145. doi: 10.1523/JNEUROSCI.3211-09.2010 20181610 PMC3669686

[ref53] Tanaka K. (1996). Inferotemporal cortex and object vision. Annu. Rev. Neurosci. 19, 109–139. doi: 10.1146/annurev.ne.19.030196.000545 8833438

[ref54] Thorat S. Proklova D. Peelen M. V. (2019). The nature of the animacy organization in human ventral temporal cortex. eLife 8:e47142. doi: 10.7554/eLife.47142.001 31496518 PMC6733573

[ref55] Toda H. Suzuki T. Sawahata H. Majima K. Kamitani Y. Hasegawa I. (2011). Simultaneous recording of ECoG and intracortical neuronal activity using a flexible multichannel electrode-mesh in visual cortex. NeuroImage 54, 203–212. doi: 10.1016/j.neuroimage.2010.08.003, PMID: 20696254

[ref56] Tsao D. (2014). The macaque face patch system: a window into object representation. Cold Spring Harb. Symp. Quant. Biol. 79, 109–114. doi: 10.1101/sqb.2014.79.024950, PMID: 25943770

[ref57] Tsutsumi S. Ushitani T. Tomonaga M. Fujita K. (2012). Infant monkeys’ concept of animacy: the role of eyes and fluffiness. Primates 53, 113–119. doi: 10.1007/s10329-011-0289-8 22143443

[ref58] Van Kerkoerle T. Self M. W. Dagnino B. Gariel-Mathis M. A. Poort J. Van Der Togt C.. (2014). Alpha and gamma oscillations characterize feedback and feedforward processing in monkey visual cortex. PNAS 111, 14332–14341. doi: 10.1073/pnas.1402773111, PMID: 25205811 PMC4210002

[ref59] Vangeneugden J. De Mazière P. A. Van Hulle M. M. Jaeggli T. Van Gool L. Vogels R. (2011). Distinct mechanisms for coding of visual actions in macaque temporal cortex. J. Neurosci. 31, 385–401. doi: 10.1523/JNEUROSCI.2703-10.2011, PMID: 21228150 PMC6623445

[ref60] Wheatley T. Milleville S. C. Martin A. (2007). Understanding animate agents distinct roles for the social network and Mirror system. Psychol. Sci. 18, 469–474. doi: 10.1111/j.1467-9280.2007.01923.x 17576256

[ref61] Whittingstall K. Logothetis N. K. (2009). Frequency-band coupling in surface EEG reflects spiking activity in monkey visual cortex. Neuron 64, 281–289. doi: 10.1016/j.neuron.2009.08.016, PMID: 19874794

[ref62] Yang Z. Freiwald W. A. (2023). Encoding of dynamic facial information in the middle dorsal face area. PNAS 120:e2212735120. doi: 10.1073/pnas.2212735120, PMID: 36787369 PMC9974491

[ref63] Yetter M. Robert S. Mammarella G. Richmond B. Eldridge M. A. G. Ungerleider L. G.. (2021). Curvilinear features are important for animate/inanimate categorization in macaques. J. Vis. 21, 3–16. doi: 10.1167/jov.21.4.3, PMID: 33798259 PMC8024783

