# Supplementary material for: Animacy processing by distributed and interconnected networks in the temporal cortex of monkeys
Source: Front Behav Neurosci. 2024 Dec 13;18:1478439. doi: 10.3389/fnbeh.2024.1478439 (PMC11671252; doi:10.3389/fnbeh.2024.1478439)
Supplement: Supplementary file 1 [file Data_Sheet_1.pdf]

Supplementary Materials

Animacy processing by distributed and interconnected networks in the temporal cortex of monkeys

Rizal Ichwansyah[1,2], Keigo Onda[1,2], Jun Egawa[2], Takeshi Matsuo[3], Takafumi Suzuki[4], Toshiyuki Someya[2], Isao Hasegawa[1], Keisuke Kawasaki[1]*

[1]Department of Neurophysiology, Niigata University School of Medicine, Niigata, Japan

[2]Department of Psychiatry, Niigata University School of Medicine, Niigata, Japan

[3]Department Neurosurgery, Tokyo Metropolitan Neurological Hospital, Tokyo, Japan

[4]Center for Information and Neural Networks, National Institute of Information and Communications Technology, Osaka, Japan

*** Correspondence:**
Keisuke Kawasaki, Ph.D.
kkawasaki@med.niigata-u.ac.jp

Keywords: categorization, animacy, inferior temporal cortex, prefrontal cortex, electrocorticography, superior temporal sulcus, Granger causality

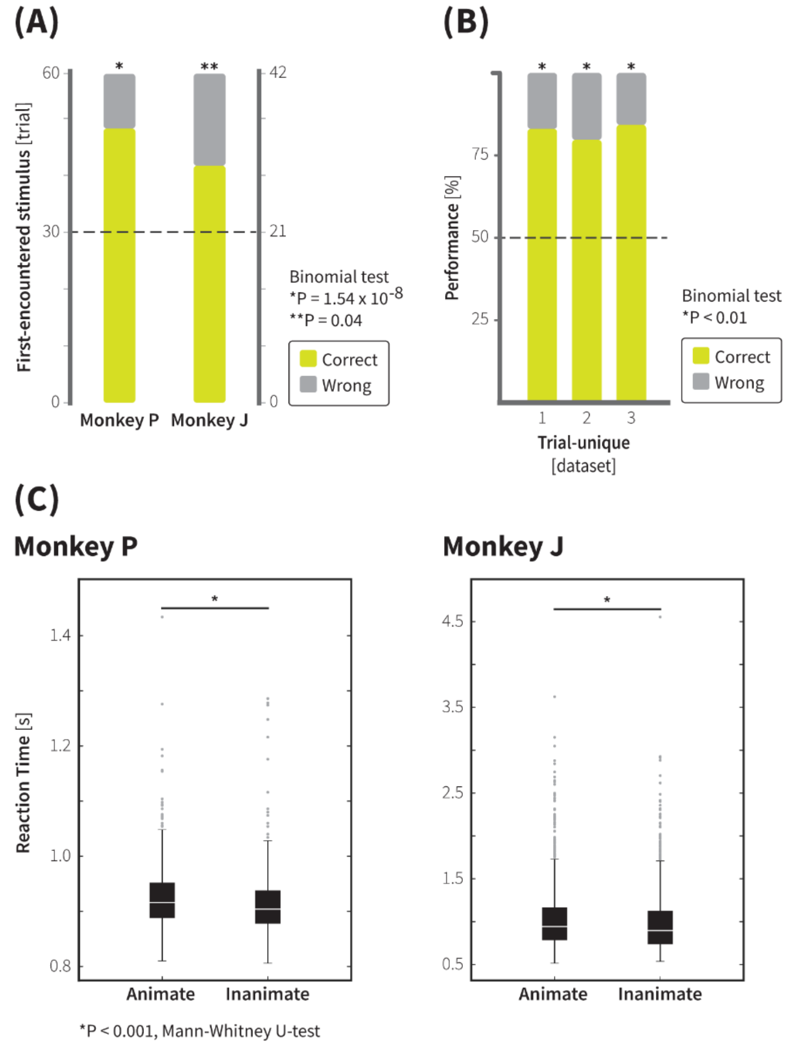

Supplementary Figure 1. Behavioral performance during training for the symbolic animate/inanimate task.
(A) Behavioral performances when the monkeys encountered brand-new stimuli. The performances were plotted on a dual y-axis graph. The left y-axis represented the number of brand-new stimulus trials performed by monkey P, while the right y-axis showed those performed by monkey J.
(B) Behavioral performance of monkey P on the unique-trial datasets. A trial-unique dataset is comprised of a high number of brand-new stimuli with fewer repetitions. Three datasets were created and tested on monkey P. A single bar represents the number of trials with correct (green) and incorrect (gray) responses. Dashed lines indicate the chance level. Significant differences are indicated by asterisk symbols ($p < 0.05$, binomial test).
(C) Monkeys' RTs to animate and inanimate stimuli. Both monkeys responded faster to inanimate movies (median RT, monkey P: 904 ms, monkey J: 896 ms) than to animate movies (monkey P: 916 ms, monkey J: 942 ms, $p < 0.001$, Mann-Whitney U test).

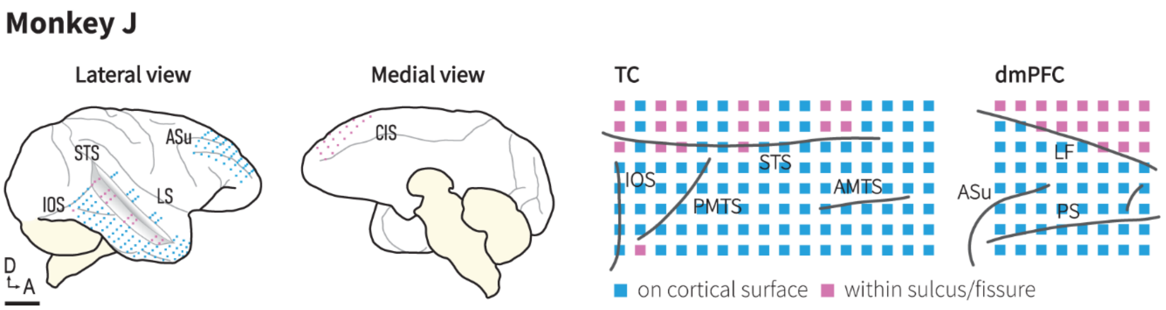

Supplementary Figure 2. ECoG electrode locations on monkey J.
Left panel: Schematic drawings of a macaque brain showing the locations of implanted electrodes in monkey J.
Right panel: Detailed location of electrodes relative to sulci and fissure (STS, superior temporal sulcus; AMTS, anterior middle temporal sulcus; IOS, inferior occipital sulcus; PMTS, posterior middle temporal sulcus; RS, rhinal sulcus; PS, principal sulcus; ASu, upper limb arcuate sulcus; CIS, cingulate sulcus; LF, longitudinal fissure). Blue squares represent electrodes implanted on cortical surfaces. Purple squares represent electrodes implanted within a sulcus or fissure. Scale bars, 10 mm.

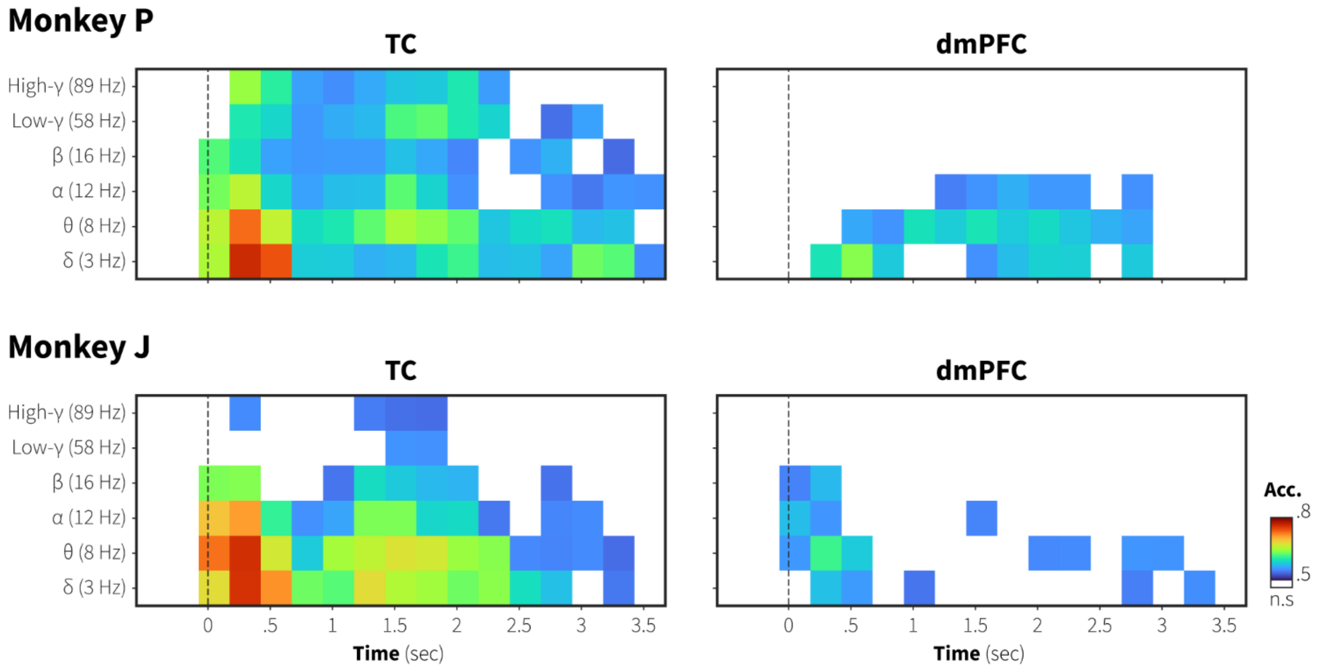

Supplementary Figure 3. Significant category performance decoded in the TC and dmPFC Category information was decoded with low and high frequency signals throughout the movie presentation in monkey P (upper) and monkey J (lower). A linear SVM was trained to decode category information using the power spectra of low time-frequency resolution ERSP. Dashed lines indicate the onset of the movie stimuli. Color scale bar indicates median decoding accuracy of bootstrap distribution. White color indicates nonsignificant, n.s., accuracy ($p > 0.05$, bootstrap test with FDR correction for 102 time-frequency points).

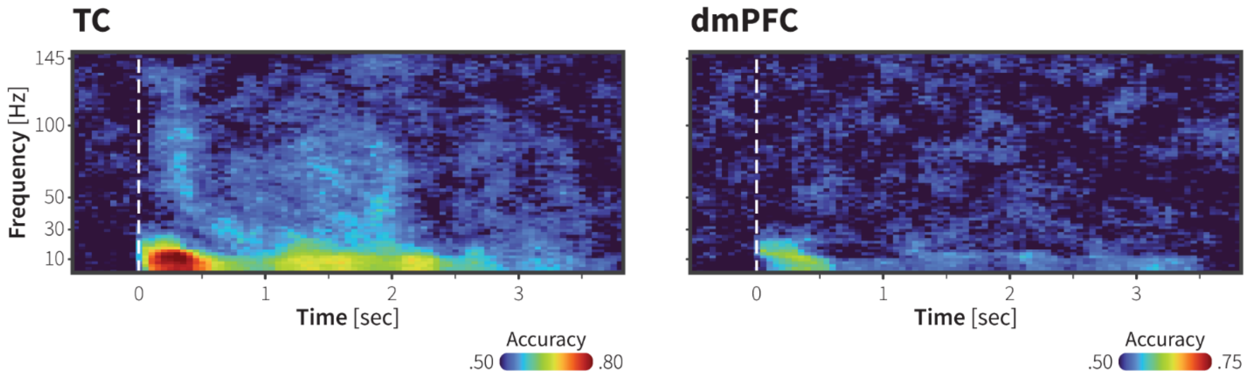

Supplementary Figure 4. Neuronal population decoding for animacy category in monkey J. Decoding for animacy-related information in the TC (upper) and dmPFC (lower) from monkey J. A linear SVM with 5-fold cross validation was trained to decode category information the high-resolution ERSP. In monkey J's dataset, there were 42 stimuli across 2109 trials (21 animate stimuli across 1055 trials). Dashed lines indicate the onset of the movie stimuli. Color scale bars indicate the population decoding accuracy of the TC and dmPFC.

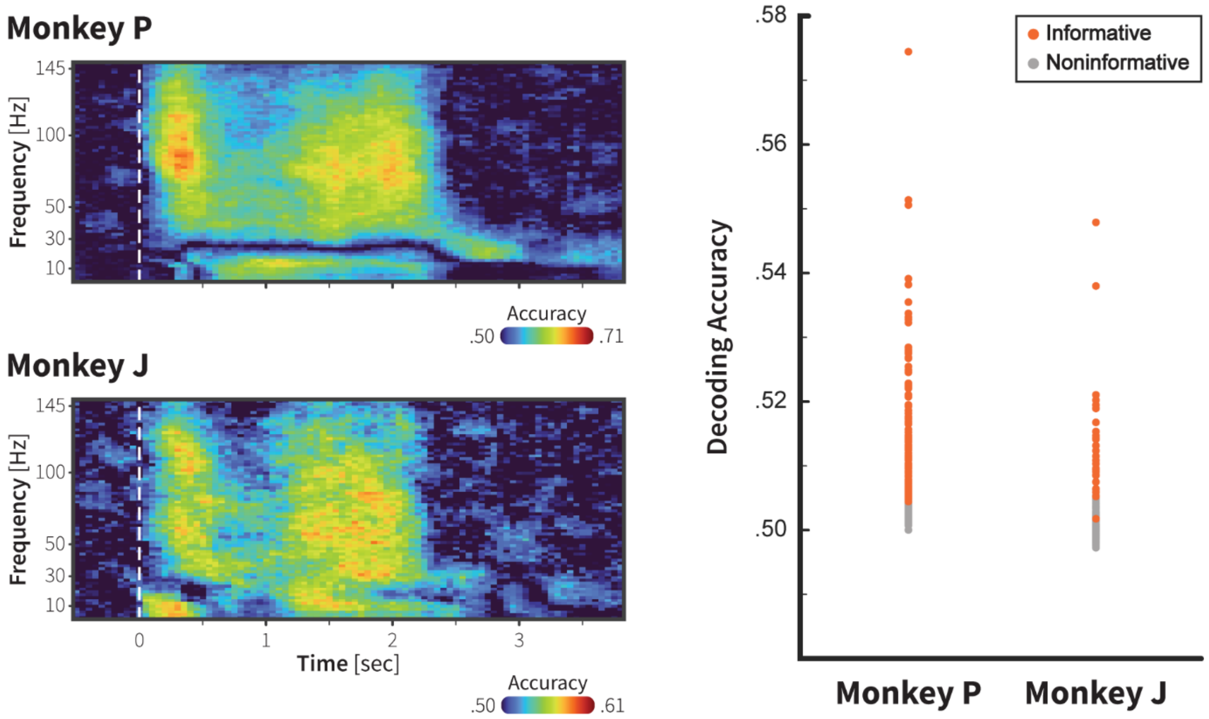

Supplementary Figure 5. Animacy category information decoded in ICs.
Left panel: an example of time-frequency decoding performance of ICs. These figures display decoding results of the most informative IC in monkey P (upper) and monkey J (lower), showing high decoding accuracies in both frequency bands. Decoding accuracy was calculated by using power spectra of power spectra of IC activation as input features. Dashed lines indicate the onset of the movie stimuli. The color scale bars represent decoding accuracy.

Right panel; informative ICs to animacy categorization. A linear SVM was employed to decode category information from low resolution time-frequency representation of IC activation. The y-axis represents average decoding performance across time-frequency dimension. Orange color dot represent informative IC ($p < 0.05$, the permutation test with Bonferroni correction for 192 ICs).

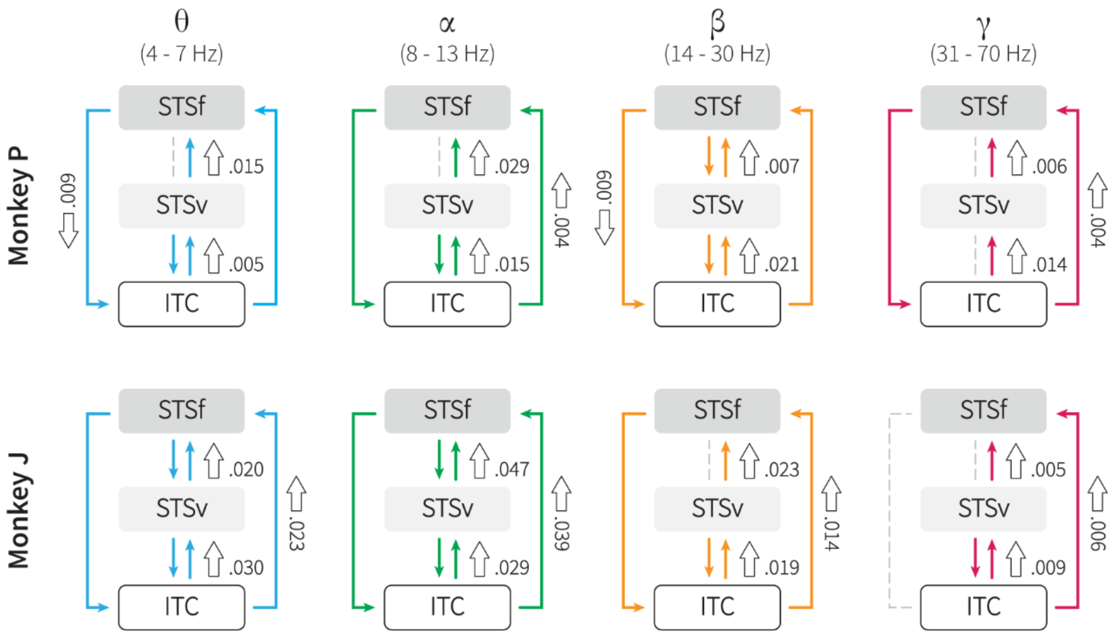

Supplementary Figure 6. GC influence in theta, alpha, beta, and gamma bands.
All statistically significant GC influences within each frequency band are summarized in block diagrams. White arrows indicate the direction of causal influence as determined by the dGC, with the value displayed next to each corresponding arrow.
